# Looking back to move forward: a twenty-year audit of herpes zoster in Asia-Pacific

**DOI:** 10.1186/s12879-017-2198-y

**Published:** 2017-03-15

**Authors:** Liang-Kung Chen, Hidenori Arai, Liang-Yu Chen, Ming-Yueh Chou, Samsuridjal Djauzi, Birong Dong, Taro Kojima, Ki Tae Kwon, Hoe Nam Leong, Edward M. F. Leung, Chih-Kuang Liang, Xiaohong Liu, Dilip Mathai, Jiun Yit Pan, Li-Ning Peng, Eduardo Rommel S. Poblete, Philip J. H. Poi, Stewart Reid, Terapong Tantawichien, Chang Won Won

**Affiliations:** 1 Center for Geriatrics and Gerontology, Taipei Veterans General Hospital, No. 201, Sec. 2, Shih-Pai Rd., Taipei, 11217 Taiwan; 2 Aging and Health Research Center, National Yang Ming University, Taipei, Taiwan; 3 National Center for Geriatrics and Gerontology, 7-340 Morioka-cho, Obu, Aichi 474-8511 Japan; 4 Center for Geriatrics and Gerontology, Kaohsiung Veterans General Hospital, No. 386 Ta-Chun 1st Rd., Kaohsiung, 81362 Taiwan; 5 Department of Internal Medicine, Faculty of Medicine, University of Indonesia, Salemba Raya No. 6, Jakarta, 10430 Indonesia; 6 The Center of Gerontology and Geriatrics, West China Medical School/West China Hospital, Sichuan University, No. 37 Guo Xue Xiang, Renmin Nan Lu, Chengdu, Sichuan 610041 China; 7 Department of Geriatric Medicine, Graduate School of Medicine, The University of Tokyo, 7-3-1, Jongo, Bunkyo-ku, Tokyo, 113-8655 Japan; 8 Division of Infectious Diseases, Daegu Fatima Hospital, 99 Ayang-ro, Dong-gu, Daegu, 710-600 Korea; 9 Rophi Clinic, 38 Irrawaddy Rd. #07-54/55, Mount Elizabeth Novena Specialist Centre, Singapore, 329563 Singapore; 10 Geriatric Medicine Centre (Healthy Ageing), Hong Kong Sanatorium and Hospital, 2 Village Rd. Happy Valley, Hong Kong S.A.R., China; 11 Division of Neurology, Department of Internal Medicine, Kaohsiung Veterans General Hospital, Kaohsiung, Taiwan; 12 Division of Geriatrics, Department of Internal Medicine, Peking Union Medical College Hospital, Beijing, 100730 China; 13 Apollo Institute of Medical Sciences and Research, Apollo Health City Campus, Jubilee Hills, Hyderabad, 500096 India; 14 National Skin Centre, 1 Mandalay Rd., Singapore, 308205 Singapore; 15 Geriatric Center, St. Luke’s Medical Center, 279 E. Rodriguez Sr. Ave., Quezon City, 1102 Philippines; 16 Division of Geriatrics, Department of Medicine, University Malaya Medical Centre, Lembah Pantai, 59100 Kuala Lumpur, Malaysia; 17 Ropata Medical Centre, Lower Hutt, 5010 New Zealand; 18 Division of Infectious Diseases, Department of Medicine, Chulalongkorn University, Bangkok, 10330 Thailand; 19 Department of Family Medicine, College of Medicine, Kyung Hee University, 1 Hoigi-dong, Dongdaemun-gu, Seoul, 130-720 Korea

**Keywords:** Asia-Pacific, Complications, Epidemiology, Healthcare burden, Herpes zoster, Immunisation, Management, Post-herpetic neuralgia, Prevention, Vaccine

## Abstract

**Background:**

Herpes zoster (HZ) is a prevalent viral disease that inflicts substantial morbidity and associated healthcare and socioeconomic burdens. Current treatments are not fully effective, especially among the most vulnerable patients. Although widely recommended, vaccination against HZ is not routine; barriers in Asia-Pacific include long-standing neglect of adult immunisation and sparse local data. To address knowledge gaps, raise awareness, and disseminate best practice, we reviewed recent data and guidelines on HZ from the Asia-Pacific region.

**Methods:**

We searched PubMed, Scopus, and World Health Organization databases for articles about HZ published from 1994 to 2014 by authors from Australia, China, Hong Kong, India, Indonesia, Japan, Korea, Malaysia, New Zealand, the Philippines, Singapore, Taiwan, Thailand, and Vietnam. We selected articles about epidemiology, burden, complications, comorbidities, management, prevention, and recommendations/guidelines. Internet searches retrieved additional HZ immunisation guidelines.

**Results:**

From 4007 retrieved articles, we screened-out 1501 duplicates and excluded 1264 extraneous articles, leaving 1242 unique articles. We found guidelines on adult immunisation from Australia, India, Indonesia, Malaysia, New Zealand, the Philippines, South Korea, and Thailand.

HZ epidemiology in Asia-Pacific is similar to elsewhere; incidence rises with age and peaks at around 70 years – lifetime risk is approximately one-third. Average incidence of 3–10/1000 person-years is rising at around 5% per year. The principal risk factors are immunosenescence and immunosuppression. HZ almost always causes pain, and post-herpetic neuralgia is its most common complication. Half or more of hospitalised HZ patients have post-herpetic neuralgia, secondary infections, or inflammatory sequelae that are occasionally fatal. These disease burdens severely diminish patients’ quality of life and incur heavy healthcare utilisation.

**Conclusions:**

Several countries have abundant data on HZ, but others, especially in South-East Asia, very few. However, Asia-Pacific countries generally lack data on HZ vaccine safety, efficacy and cost-effectiveness. Physicians treating HZ and its complications in Asia-Pacific face familiar challenges but, with a vast aged population, Asia bears a unique and growing burden of disease. Given the strong rationale for prevention, most adult immunisation guidelines include HZ vaccine, yet it remains underused. We urge all stakeholders to give higher priority to adult immunisation in general and HZ in particular.

**Electronic supplementary material:**

The online version of this article (doi:10.1186/s12879-017-2198-y) contains supplementary material, which is available to authorized users.

## Background

Herpes zoster (HZ) is a prevalent and debilitating viral disease that often causes serious complications and proves challenging to treat. Consequently, HZ results in substantial morbidity, healthcare expenditure, loss of productivity, and diminished quality of life (QoL). Older people bear the greatest burden of disease, which is increasing as populations age. Despite a strong rationale for prevention, availability of an effective vaccine, and guidelines recommending HZ immunisation, vaccination has not become routine practice. One reason was limited availability of HZ vaccine after its launch in 2006 [1, 2]. Although an ample supply was restored, a fundamental barrier in Asia is long-standing neglect of preventive adult healthcare. Since Ilina Isahak highlighted this issue in 2000 [3], progress been limited and adult immunisation is still not given the priority that it merits [4, 5, 6]. Moreover, existing HZ immunisation guidelines are based on evidence from Western populations, which creates a perceptual barrier to changing management practices in Asia. Conversely, more locally-relevant data may promote guideline implementation; however, many Asian countries lack such data [7]. To address these concerns, we systematically reviewed literature on HZ from the Asia-Pacific region. Our objectives were: 1) To provide a comprehensive overview of the epidemiology, burden, and current management of HZ; 2) To disseminate best practice in HZ immunisation; and 3) To provide an up-to-date source of reference and information for stakeholders concerned with reducing the burden of HZ in Asia-Pacific. This review summarises our key findings and recommendations.

## Methodology

### Literature search strategy

We reviewed literature on HZ published over 21 years by authors from 14 Asia-Pacific countries. We searched three databases: PubMed (United States [US] National Library of Medicine National Institutes of Health), Scopus (Elsevier), and the World Health Organization (WHO) Global Health Library Regional Indexes. PubMed and Scopus searches used the search term ‘zoster’ in title, abstract, and author keywords fields, AND the term ‘country name’ in the affiliation field. The countries/regions were: Australia, China, Hong Kong, India, Indonesia, Japan, Korea, Malaysia, New Zealand, the Philippines, Singapore, Taiwan, Thailand, and Vietnam OR Viet Nam. WHO searches used the terms ‘country name’ AND ‘zoster’. Searches were limited to articles with abstracts in English published electronically since 1 January 1994 and before 31 December 2014; alerts were set to capture eligible articles added to PubMed and Scopus after the initial search dates.

We performed separate internet searches for guidelines on HZ immunisation. These used the country names and also vernacular terms for their inhabitants, eg, Thai, Filipino, Malay, combined with the terms herpes zoster, guidelines, recommendations, adult immunisation, vaccine, vaccination.

#### Data management

PubMed and Scopus results were downloaded in Comma Separated Value format then saved to Microsoft Excel files. WHO Global Health Library searches were exported as text files then imported into Microsoft Excel. Results from each database were screened to remove duplicates before entering the results for each country into a Microsoft Excel workbook. Duplicates between databases were identified by using conditional formatting to highlight entries with the same title. Duplicates between countries were identified similarly, by highlighting entries with the same database identity codes.

#### Inclusion and inclusion criteria

We included articles about four topics: epidemiology and burden; complications and comorbidities; management and prevention; and recommendations/guidelines. We excluded articles about: subjects only younger than 18, or from another country; primary varicella zoster virus (VZV) infection or not specifying that VZV infection was reactivated; negative disease associations, cases of coincidental HZ, or diseases other than HZ; extraneous research topics, eg, basic/molecular/experimental, diagnosis, technology; or reviews without country-specific information. Miscellaneous exclusions were editorials, correspondence or errata concerning included articles; study protocols, and articles on terminology, hypotheses, non-human subjects, or knowledge, attitudes and behaviour relating to HZ and its treatment.

## Literature search results

**Fig. 1:**
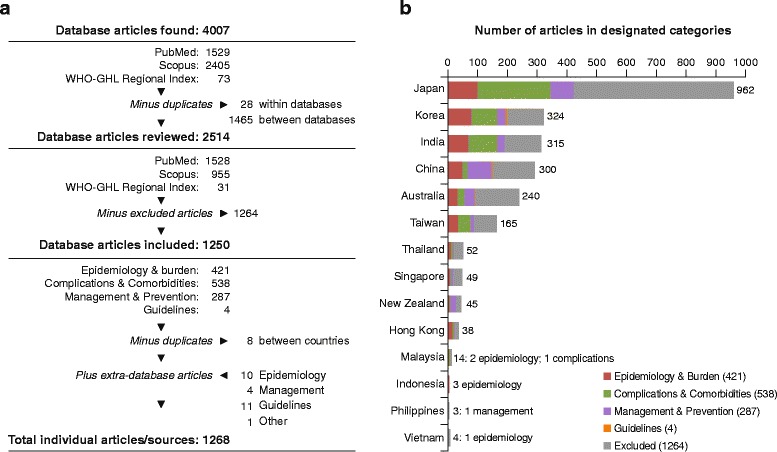
**a** Literature search and selection; **b**) Included articles by country and topic. WHO-GHL, World Health Organization Global Health Library

The database searches returned 4007 articles (Fig. 1a). Having excluded 1493 duplicates and 1264 non-relevant articles, 1250 were assigned to four categories: epidemiology and burden (421); complications and comorbidities (538); management and prevention (287); and guidelines (4) (Fig. 1b). The separate search for guidelines found recommendations on adult immunisation from Australia, India, Indonesia, Malaysia, New Zealand, the Philippines, South Korea, Taiwan, and Thailand.

‘Grey literature’ included other key studies/data cited in retrieved articles or not stored in the searched databases, eg, national statistics or reports published locally; 16 such sources were added.

## Clinicopathology

**Fig. 2:**
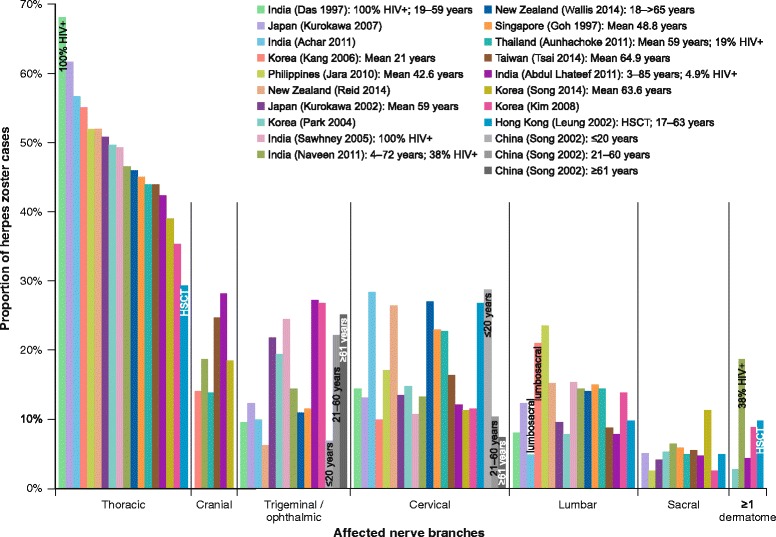
Nerve branches affected by herpes zoster. HIV+, human immunodeficiency virus positive; HSCT, haematopoietic stem cell transplant

Herpes zoster is caused by reactivation of VZV dormant in nerve root ganglia since a primary varicella infection (Chickenpox). The cardinal symptom is neuropathic pain, often accompanied by a self-limiting vesicular rash and/or inflammation disclosing the source nerve [8–10]. In Asia, as elsewhere, the classic presentation is unilateral HZ affecting a single thoracic dermatome, although HZ frequently involves the trigeminal, cranial, or cervical nerves; lumbosacral HZ is less common (Fig. 2) [11–31]. The presentations and course of HZ also vary depending on patients’ age, health, and immune status. Older people are more likely to have trigeminal VZV reactivation and worse and longer-lasting rash and pain [20, 32], whereas immunocompromised patients tend to have relatively more thoracic HZ [14, 33]. All physicians should therefore beware that HZ assumes an array of guises.

### Atypical HZ

Reactivated VZV can affect any part of the body. Ocular symptoms due to involvement of the ophthalmic division of the trigeminal nerve are common [34]; however, the maxillary and mandibular branches may occasionally be affected, resulting in oro-cutaneous manifestations [35, 36]. Atypical presentations usually reflect underlying immunocompromise, and other unusual locations have included the eardrum [37], genitalia [38, 39], papilla [40], and finger [41].

#### Zoster sine herpete

VZV reactivation does not always manifest cutaneously. Zoster “sine herpete” causes unexplained pathologies, including neuralgia [42], ocular [43], facial [44], or neuromotor palsy or paralysis [45, 46], and cerebral or ocular inflammations [47, 48].

#### Multidermatomal, disseminated and visceral HZ

Rarely, especially in patients who are elderly or otherwise immunocompromised, HZ involves two or more distinct dermatomes [49, 50], spreads across multiple sites [51, 52], appears as a generalised rash [53, 54], or worse, affects internal organs [9, 10]. Although occasionally seen in immunocompetent individuals [55–64], such unusual presentations constitute fewer than 1% of total cases, being particularly characteristic of iatrogenic immunosuppression in transplant recipients [65, 66] or cancer patients [67–71], or human immunodeficiency virus (HIV) infection, in which bilateral [72, 73], multidermatomal [74], disseminated [75], or recurrent [73, 76, 77] HZ are often the presenting symptom [72, 78–80].

Disseminated HZ predicts higher risk of complications [81] and may portend abdominal HZ, which has high mortality despite antiviral therapy, especially in profoundly immunocompromised patients [9, 10, 82]. For example, haematopoietic stem cell transplant (HSCT) recipients have died of fulminant VZV hepatitis [83, 84]. Such patients may present with severe abdominal pain [82, 85–87] either sine herpete or before lesions appear [9, 10]. Besides hepatitis, viscerally disseminated HZ may also cause pancreatitis [88, 89], colitis [90], pneumonitis [91, 92], or pneumonia [69, 93], which causes most fatalities [10].

### Pain

Irrespective of its outward appearance, pain is the hallmark of HZ in adulthood. Acute pain and post-herpetic neuralgia (PHN) are its most unbearable and debilitating symptoms and severely impair QoL and everyday activities [15, 18, 20, 30]. Pain is notoriously challenging to manage, especially once established, making this the most compelling reason for early intervention [9, 25, 94, 95].

Almost all adult HZ patients suffer pain, which can be excruciating and is often described as the worst ever experienced [10, 96]. Many cases are presaged by prodromal neuralgia or malaise, with abnormal sensations such as itching, paraesthesia and hyperaesthesia [10, 18]. In India, Korea, Singapore and Taiwan, around two-thirds of patients reported prodromal pain, which more than 90% rated moderate-to-severe [18, 20, 25, 31]. Among 150 Taiwanese patients, 98% had acute pain at enrolment (87% moderate-to-severe) [20]; similarly, 95% of Indian and Singaporean patients experienced pain during the course of disease [12, 18]. Pain is more frequent and severe in older patients and can have devastating impacts; patients feel anxious and miserable, have disturbed sleep and cannot work normally or enjoy life [10, 12, 15, 18, 20, 25, 30]. Besides age, the severity and duration of acute herpetic pain correlates with the degree of prodromal pain, the severity of skin lesions, trigeminal/ophthalmic involvement, anxiety or depression, and comorbid disease [23, 32, 33, 97–100].

## Epidemiology and risk factors

### Seroepidemiology

**Fig. 3:**
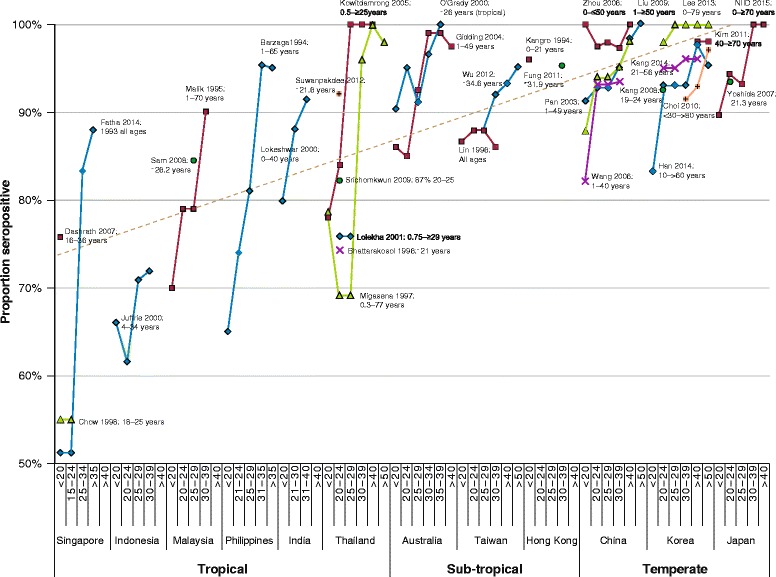
Ages of varicella zoster virus seroconversion among adults^a^ in tropical, subtropical and temperate Asia-Pacific countries. NIID, National Institute of Infectious Diseases, Japan. ^a^ Reported seroprevalence in age-groups including individuals ≥18 years old

The aetiologic prerequisite of HZ is prior VZV infection, which most people contract in childhood, unless vaccinated preemptively [2, 7, 101, 102]. Although acquisition may be delayed in tropical climes, around 90% of adults age 30–39 in Asia-Pacific countries/regions have seroconverted, with almost all aged ≥40 years VZV seropositive and therefore at risk of HZ (Fig. 3) [7, 103–135].

### Incidence

**Fig. 4:**
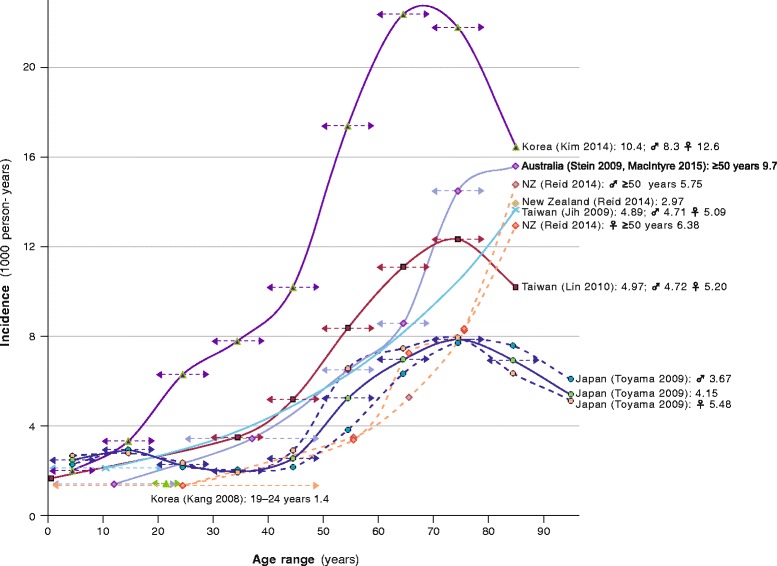
Age-specific incidence of herpes zoster in Asia-Pacific countries. NZ, New Zealand; ♀, female; ♂, male

HZ is very common in the Asia-Pacific region. Alike Western populations, estimated lifetime risk is approximately one-third [10, 136], and incidence of 3–10/1000 person-years (PY) rises steeply above age 40 and peaks between 70–80 years (Table 1, Fig. 4); there is female predominance in diverse Asia-Pacific populations, especially between ages 50–70 [11, 17, 26, 104, 126, 132, 133, 136–144]. Authors ascribe higher age-specific incidence rates in Korea than other countries, to heavy use of state-insured healthcare by patients with mild HZ symptoms, besides being based on data from recent years during which HZ incidence has risen steeply [138, 140]; on the other hand, much lower incidence in Thailand [30, 144] probably reflects under-reporting.

**Table 1:** Herpes zoster incidence rates in Asia-Pacific

Country (source)	Study year(s)	HZ cases	Age range (years)	Incidence (cases/1000 person years)											
				Overall	By sex		Age-range (years)								
					Male	Female	<10	10–19	20–29	30–39	40–49	50–59	60–69	70–79	>80
Australia (MacIntyre 2003) [102]	1999	59200	All ages	8.3^a^											
Australia (Stein 2009) [104](MacIntyre 2015) [807]	1998–2005	379	≥50	9.7^a^			1.4 (<25)			3.4 (25–49)		6.5	8.6	14.5	15.6
Japan (Toyama 2009) [139]	1997–2006	48388	0– ≥ 90	4.2	3.7	4.6*	2.5	2.9	2.3	2.0	2.5	5.2	7.0	7.8	6.9^b^
New Zealand (Reid 2014) [17]	2009–2013	339	All ages	3.0	5.8^c^	6.4^c^	1.3 (≤50)								13.9
Korea (Kim YJ 2014) [138]	2011	529690	0– ≥ 80	10.4	8.3	12.6	2.0	3.3	6.3	7.8	10.2	17.4	22.4	21.8	16.5
Korea (Kang 2008) [129]	2004–2005	705	19–24	1.4				1.4 (19–24)							
Korea (Choi 2010) [130]	2003–2007	2431744	All ages	10.0^d^											
Korea (Park SY 2004) [26]	1994–2003	1089	All ages	3.0											
Thailand (Aunhachoke 2011) [30]	2008	180	≥50	0.3^e^											
Taiwan (Wu PY 2013) [141]	2000–2009		All ages	6.2											
Taiwan (Wu CY 2010) [142]	2000–2005	24527	20– ≥ 60	7.0											
Taiwan (Chao DY 2012) [143]	2004–2008	7574	All ages	5.7											
Taiwan (Jih 2009) [137]	2000–2006	34280	1– > 80	4.9	4.7	5.1	2.1								13.7
Taiwan (Lin 2010) [136]	2000–2005	672782	≤1– ≥ 80	5.0	4.7	5.2*	1.6			3.5	5.2	8.4	11.1	12.3	10.2
Median (range)				5.0^f^ (0.3–10.4)	4.7 (3.7–8.3)	5.2 (4.6–12.6)	2.0 (1.6–2.5)	3.1 (2.9–3.3)	4.3 (2.3–6.3)	3.5 (2.0–7.8)	5.2 (2.5–10.2)	7.4 (5.2–17.4)	9.8 (7.0–22.4)	13.4 (7.8–21.8)	13.8 (6.9–16.5)
Quartile range (IQR)				3.6–7.3 (3.7)	4.7–5.8 (1.0)	5.1–6.4 (1.3)	1.9–2.2 (0.3)	3.0–3.2 (0.2)	3.3–5.3 (2.0)	2.7–5.7 (2.9)	3.9–7.7 (3.8)	6.8–12.9 (6.1)	9.0–16.8 (7.7)	10.1–17.1 (7.0)	10.2–13.9 (3.7)

*HZ* Herpes zoster, *IQR* Interquartile range

^a^National estimate extrapolated from sample

^b^80–89

^c^Age >50 years

^d^Clinic visits for herpes zoster

^e^ Based on reported cases per 100000 population

^f^Subjects of all ages

*P < 0.001

Bold data values signify significant difference between incidence rate in females vs. males

**Fig. 5:**
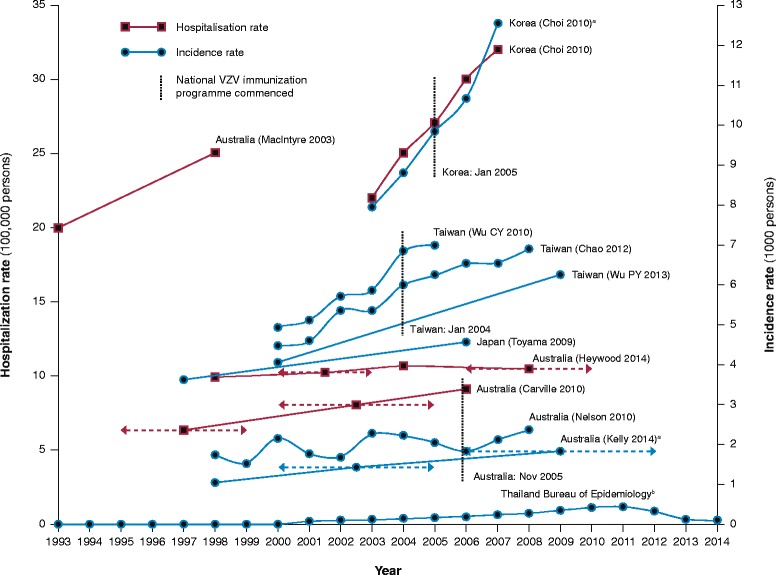
Trends in herpes zoster rates in Asia-Pacific countries. ^a^ Consultations. ^b^ Reported cases

As in other populations, HZ incidence rates are increasing in countries across Asia-Pacific (Table 2, Fig. 5) [2, 102, 139–150]; the principal cause is most likely rising incidence in ageing populations, especially among women, together with growing prevalence of chronic diseases and use of immunosuppressive medications [2, 102, 139–141, 143, 147]. HZ incidence in Taiwan rose by 20% from 2004–05 to 2006–08 despite remaining stable in 10–49 year-olds, with a significant increase in older people [143]. Hypothetically, mass varicella immunisation might also contribute to this trend; if natural exposure to VZV strengthens immunity to HZ – exogenous boosting – HZ incidence in countries that institute routine childhood varicella immunisation (Table 3) would be expected to rise subsequently among the unvaccinated population, due to declining prevalence of varicella [2, 141, 146, 151, 152]. Although there is some evidence that exogenous boosting does occur [151], there is very little for a substantive contribution to HZ, and its role, if any, in HZ epidemiology remains obscure [2, 141, 143, 146, 147, 151, 152]. Rising HZ rates predating mass varicella immunization (Fig. 5) suggest that other factors are more important; besides those already mentioned, these may also include changes in health-seeking behaviour and more comprehensive and accurate disease surveillance [151, 152].

**Table 2:** Trends in herpes zoster incidence, hospitalisation and healthcare costs in Asia-Pacific countries

Country (source)	Metric	Years compared	Herpes zoster rate	Annual increase (%)
Australia (Kelly 2014) [146]	Cases/1000 consultations	1998 2006–12	1.03 1.81	6.3
Australia (Nelson 2010) [145]	Management/1000 GP consultations	1998 2008	1.71 2.35	3.4
Australia (MacIntyre 2003) [102]	Hospitalisations/100000 population	1993 1998	20 25	4.2
Australia (Carville 2010) [147]	Hospitalisations/100000 population^a^	1995–99 2006–07	6.3 9.1	3.1
Australia (Heywood 2014) [2]	Hospitalisations/100000 population	1998 2004 2006–10	9.2 10.6 10.4	2.2 −0.4
Korea (Choi 2010) [130]	Hospitalisations/1000 population	2003 2007	0.22 0.32	9.1
	Consultations/1000 population	2003 2007	7.93 12.54	11.6
	Total socioeconomic cost (US$)	2003 2007	75,921,348 143,774,888	17.9
Japan (Toyama 2009) [139]	Incidence/1000 person-years	1997 2006	3.61 4.55	2.6
Taiwan (Lin 2010) [136]	Healthcare cost (NT$)	2000 2004	250,000,000 319,000,000	5.5
Taiwan (Wu CY 2010) [142]	Incidence/1000 population	2000 2005	4.94 7.00	7.0
Taiwan (Chao DY 2012) [143]	Incidence/1000 population	2000 2008	4.45 6.89	6.1
Taiwan (Wu PY 2013) [141]	Incidence/1000 person-years	2000 2009	4.40 6.24	5.5
Thailand (Bureau of Epidemiology)	Reported cases/100000 population	2001 2010 2014	6.44 40.49 7.65	52.9 −8.1
Median (range)				5.5 (−8.1–17.9)
Quartile range (IQR)				3.0–7.5 (4.6)

*GP* General practitioner, *IQR* Interquartile range

^a^Principal diagnosis of herpes zoster

**Table 3:** Asia-Pacific countries with national varicella immunisation programmes

Country	Varicella vaccine schedule		Planned extent of coverage	Programme commenced (month, year)
	Dose(s)	Age		
Australia	1	18 months, with catch-up at 10–13 years^a^	Entire population	November 2005
Hong Kong	2	12 months & 6 years^b^	Entire population	November 2005
Japan	2	12–36 months	Entire population (voluntary category)^c^	October 2014
New Zealand	1	15 months, with catch-up at 11 years^d^	Entire population	July 2017
South Korea	1	12–15 months	Entire population	January 2005
Taiwan	1	12–18 months	Entire population	January 2004

^a^Schools programme

^b^Primary 1 school age

^c^Not covered under the Preventive Vaccinations Act for Routine Vaccination against Category A Diseases

^d^For non-vaccinated children who have not already had a varicella infection

#### Recurrence

Reported HZ recurrence rates range from 2.3% to 8.0% overall [13, 153, 154] and are higher in women, immunocompromised patients, and individuals aged 50–70 or with PHN [153].

### Risk factors and comorbidities

**Fig. 6:**
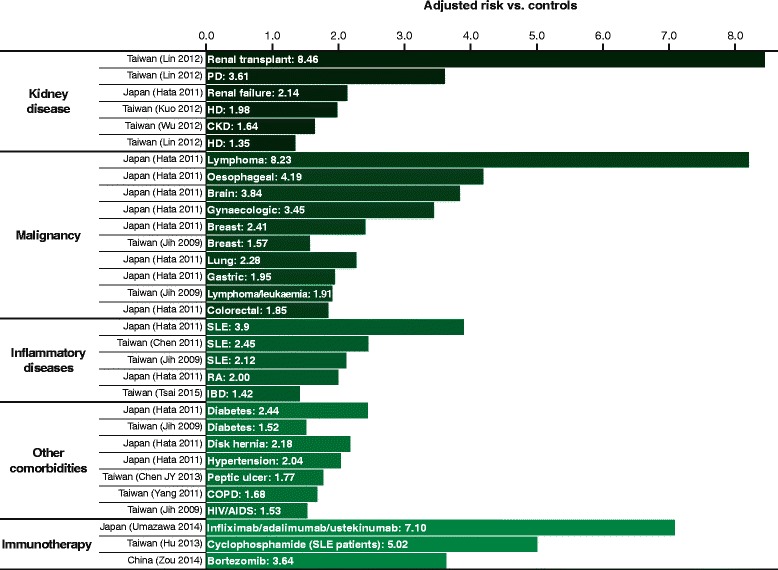
Relative risk of herpes zoster in different predisposing conditions. PD, peritoneal dialysis; HD, haemodialysis; CKD, chronic kidney disease; SLE, systemic lupus erythematosus; RA, rheumatoid arthritis; IBD, inflammatory bowel disease; COPD, chronic obstructive pulmonary disease; HIV/AIDS, human immunodeficiency virus/acquired immunodeficiency syndrome

The constitutional cause of HZ is failing cell-mediated immunity (CMI) that becomes too weakened to suppress latent VZV [155]. CMI wanes naturally with age [156], explaining why advanced age is the preeminent risk factor for HZ and PHN among all populations [10, 95]. Besides immune senescence, other conditions that diminish CMI likewise increase the risk of HZ. The principal predisposing factors are iatrogenic or pathologic immunosuppression (Fig. 6) [137, 157–167]. Inpatients with severe illnesses or recovering from surgery develop HZ more frequently than others [168].

#### Immunosuppressant therapies

##### Transplantation

Organ, tissue or cell transplant recipients require immunosuppressive therapy with cyclosporine and/or other drugs that prevent transplant rejection. Consequently, they often develop HZ or other opportunistic infections, especially within the first year following transplantation [16, 66, 169–180]. HZ rates of up to >80% have been reported [170, 181], and transplant recipients are especially prone to disseminated and visceral HZ with potentially fatal inflammatory complications [70, 82, 181–186].

#### Anti-inflammatory and cytotoxic agents

##### Corticosteroids

Systemic glucocorticosteroids are immunosuppressant and have been associated with HZ rates above 30% in long-term use [187–189].

##### Cyclophosphamide and others

Cyclophosphamide-based and other chemotherapeutic regimens substantially increase the risk of HZ in patients with lymphoma [190–192]. High rates have also been reported in leukaemia patients treated with arsenic trioxide [193–196]. Systemic lupus erythematosus (SLE) and lupus nephritis are also treated with cyclophosphamide, often combined sequentially with azathioprine and prednisolone or mycophenolate mofetil. HZ is the most common drug-related infection, with rates of up to 40% [166, 197–201].

HZ is also an adverse effect of azathioprine and mycophenolate mofetil in SLE and other therapeutic settings [202–214]. Cases of disseminated HZ have occurred in rheumatoid arthritis patients treated with leflunomide, with increased risk in those taking concomitant methotrexate and corticosteroids [215, 216]. HZ was the most common infection in leflunomide-treated lupus nephritis [217]. The multiple sclerosis drug cladribine is associated with an increased rate of HZ versus placebo [218].

##### Biologic agents

Antibody therapies for cancer, transplant rejection and other conditions are associated with elevated rates of VZV reactivation, both alone and combined with other biologics or chemotherapies. These include alemtuzumab [219], rituximab [220–224], and abatacept [225]. Tofacitinib is associated with significantly higher rates HZ in Asians than other races [226, 227]. Increased relative risk of HZ has also been reported with ustekinumab, adalimumab and infliximab [165, 228–230].

***Bortezomib*** Bortezomib directly impairs CMI and is an independent risk factor for HZ [166], with incidence of up to 40% in some Asia-Pacific studies [231–250]. HZ prophylaxis is advisable before commencing such therapy [251].

#### Other iatrogenic risk factors

##### Trauma

Accidental or iatrogenic trauma can trigger HZ [252–258]. This is particularly associated with ocular, facial or dental surgeries [259–268], and heightened vigilance is warranted following such procedures, particularly in elderly or immunocompromised patients. Ironically, neurosurgical treatment of PHN occasionally causes HZ [269–271].

##### Anaesthesia

General anaesthesia may compromise immune function, and mothers anaesthetised for caesarean delivery have a small increased risk of HZ during the following year [272].

##### Statin therapy

Statin users have slightly increased risk of HZ, likely due to statin-mediated immunosuppression [273, 274].

##### Drug-induced hypersensitivity syndrome

Drug-induced hypersensitivity is a severe reaction to certain drugs that involves the reactivation of herpes viruses, including VZV. Systemic corticosteroids may contribute to this risk, and like immune restoration syndrome, withdrawal of corticosteroid may trigger HZ [275].

#### Infections

##### HIV/AIDS

HIV/Acquired Immunodeficiency Syndrome (AIDS) is a major healthcare problem in Asia. With prevalence of up to 30% and incidence as high as 40–50/1000 PY [276–303], HZ is so common in HIV-infected individuals that it may be a pragmatic prognostic marker [14, 27, 28, 76], especially among younger individuals [304–309]. AIDS patients may have recurrent [73, 76, 77, 80], disseminated [27, 78], or cerebral HZ infections [310, 311]. HZ incidence correlates with low CD4+ cell count [310, 312–314] and may be significantly reduced by highly-active antiretroviral therapy (HAART); however rates in the post-HAART era remain double-digit [281, 303].

***Immune reconstitution syndrome*** Paradoxically, patients regaining immunocompetence following HAART are at higher risk of HZ than when immunodeficient, probably due to increasing CD8 cell count, a phenomenon termed immune reconstitution inflammatory syndrome [315–318].

##### Varicella zoster virus

Counterintuitively, Taiwanese healthcare workers frequently exposed to VZV did not gain enhanced immunological protection, and had higher incidence of HZ than the general population; however, this may be because their very stressful working environment dampened CMI, overriding any protective effect [142]. Epidemiology data from Japan [319] and elsewhere suggests that varicella exposure boosts immunity to HZ [102, 151]. Nevertheless, rare localised HZ clusters suggest that VZV re-exposure might occasionally provoke recrudescent HZ; the mechanism is unknown, but may involve disruption of CMI by the humoral response to VZV reinfection [320]. Although VZV transmission from patients with HZ is rare, due to less and more localised viremia, exudate from open HZ lesions may potentially infect close contacts who have not had varicella or been immunised [10, 320–324].

##### Other pathogens

Rare cases of HZ associated with tuberculosis or malaria have been reported in China and India [325–328].

#### Intrinsic risk factors

##### Pregnancy

Pregnancy is another immunosuppressed state that may potentiate HZ [120]. Although this seldom occurs, HZ is the most common skin infection that dermatologists encounter in pregnant women, accounting for up to 30% of cases [329]. While challenging to treat, unlike congenital varicella, HZ poses minimal risk to the foetus [330, 331].

##### Good’s syndrome

Good’s syndrome is a very rare immunodeficiency disease predisposing to HZ [332].

##### Nutritional deficiency

Micronutrient deficiencies that diminish CMI may increase susceptibility to HZ and PHN; supplements, particularly vitamin D, can boost VZV immunity [333–335].

##### Genetic predisposition

Asians have genetic predispositions to HZ; specifically, human leukocyte antigen gene polymorphisms have been linked to VZV reactivation in East Asian populations [336–338].

#### Associated diseases

Unsurprisingly, many HZ patients have common diseases of older age (Table 4), most frequently hyper-tension, diabetes, malignancies, and pulmonary disorders [11, 12, 15, 23, 26, 28, 137, 154, 166, 339]; however, since comorbidities and/or complications account for high proportion of hospitalised HZ cases [11, 12, 15, 23, 26, 28, 102, 104, 136, 137, 154, 166, 340], these may be more prevalent among such patients than in the general population.

**Table 4:** Common comorbidities of herpes zoster in Asia-Pacific patient populations

Country (source)	Setting (patient number)	Age profile (years)	Proportions with comorbidities (%)					
			Any	Hypertension	Diabetes	Malignancy	Pulmonary	Cardiovascular
Korea (Park SY 2004) [26]	Dermatology (1089)	NA	36.5	12.6	7.9	0.6	1.7^a^	1.6^c^
Korea (Kim YM 2008) [11]	Dermatology (297)	NA	56.2	32.1	17.5	10.4	7.5^a^	NA
China (Yuan LL 2014) [339]	Hospitals (2960)	Median 60		22.2	11.7		6.2^b^	10.7
Philippines (Jara 2010) [15]	Hospital (221)	Mean 43	28.0	13.6		1.8	8.1	2.7
Japan (Kurokawa 2002) [23]	Hospitals (263)	Mean 59	41.1		9.6	13.7		
Thailand (Tunsuriyawong 2005) [154]	Hospital (339)	Mean 59	26.8	4.0	2.5	1.0		
India (Abdul Latheef 2011) [12]	Hospital (205)	47% >40	14.6		5.3	9.2		
Taiwan (Jih 2009) [137]	National (34380)	33% >60			20.6	2.7		
Taiwan (Hu 2013) [166]	Hospital (2306)		24.3			14.8		
Taiwan (Wu CY 2010) [142]	National (702932)					1.0		
Japan (Kurokawa 2007) [28]	Dermatology (316)		42.1	4.1	6.6	15.2		
		Median (range)	32.3 (14.6–56.2	13.1 (4.0–32.1	8.8 (2.5–20.6)	6.0 (0.6–14.8)	6.9 (1.7–8.1)	2.7 (1.6–10.7)
		Quartile range (IQR)	26.2–41.4 (15.2)	6.2–20.1 (13.8)	6.3–13.2 (6.9)	1.2–12.9 (11.7)	5.1–7.7 (2.6)	2.2–6.7 (4.6)

*NA* Data not available, *IQR* Interquartile range

^a^Asthma

^b^Respiratory infections

^c^Angina pectoris

Patients with certain underlying diseases have apparently increased risk of HZ; however, it may be difficult to ascertain whether such relationships are causal, reflect underlying pathology, or are coincidental. In cancer, SLE, and other diseases treated with immunosuppressants, HZ may be largely treatment-related, although the disease too may make an indeterminate contribution to increasing the risk [10]. In others, for example, diabetes and renal disease, impaired CMI may be a common factor. Adjusted for age and other confounding variables, several comorbidities have been identified as independent risk factors for HZ, most notably renal disease, malignancies, and SLE (Fig. 6) [137, 157–167].

##### Renal disease

The incidence of HZ increases across the spectrum of renal disease, with transplant patients at highest risk followed by peritoneal dialysis and haemodialysis [157, 159, 160, 341–343]. The risk of HZ in haemodialysis is heightened by corticosteroid use, but may be mitigated by iron and vitamin D supplementation [334, 335].

##### Inflammatory autoimmune diseases

HZ frequently afflicts patients with connective tissue disorders [344], with double the risk in rheumatoid arthritis [158]. Patients with SLE have impaired CMI and are treated with immunosuppressant anti-inflammatory drugs, which significantly increases their risk of HZ [137, 161, 345, 346], particularly if they have neuropsychiatric manifestations, renal involvement, or receive cyclophosphamide [166]. Likewise, increased risk of HZ associated with inflammatory bowel disease probably reflects iatrogenic immunosuppression in many patients [162, 347].

##### Cancer

Cancer patients may be immunosuppressed due to malignancy, its treatment, or both [12, 39, 348]. Risk of HZ is increased in both solid and haematologic cancers [158, 349, 350], especially leukaemia and lymphoma [137, 158].

##### Pulmonary disease

Both immune dysregulation and corticosteroid therapy may account for increased risk of HZ in chronic obstructive pulmonary disease. Patients taking oral corticosteroids are at the greatest risk [164].

##### Diabetes

Patients with diabetes have impaired VZV-specific CMI [351]; however, evidence that diabetes is a risk factor for HZ [137, 158, 352] is inconclusive.

##### Peptic ulcer disease

Peptic ulcer disease independently predicts HZ, having excluded confounding effects of anti-inflammatory drugs and *Helicobacter pylori* infection [163].

##### Psychiatric disorders

CMI is also diminished in psychiatric disorders. The risk of HZ is increased in patients with affective psychoses, neurotic illness, personality disorders and other mental disorders, especially among those younger than 60 [353].

## Complicating presentations and disease associations

### Post-herpetic neuralgia

The most common complication of HZ in adults everywhere is pain outlasting the eruptive phase [94]. Definitions of PHN are inconsistent, ranging from ≥1 to ≥6 months since rash onset; however, PHN may persist for years [10]. Reported rates vary widely, depending on patients’ age profiles and the definitions used [20]; nonetheless, the incidence generally ranges between ≤10 to ≥25%, with up to 20% still enduring pain after 6 months (Table 5) [11, 12, 15, 18, 20, 21, 23, 25, 26, 28, 30, 96, 104, 130, 137, 154, 340, 354–359]. PHN correlates strongly with advancing age [15, 18, 21, 96, 102, 137, 340, 356, 357], the other major prognostic factors being the severity of HZ and intensity of acute pain [23, 99, 137, 356, 357, 360–362]. Comorbid risk factors include diabetes, lymphoma/leukaemia, SLE [137, 363], peptic ulcer disease [364], and micro-nutrient deficiencies [333]. There is also evidence of specific phenotypic [358] and genotypic predispositions [365–368]. Bortezomib may aggravate PHN independently of VZV reactivation [369].

**Table 5:** Herpes zoster complication rates in Asia-Pacific patients

Country (source)	Setting (number of patients)	Age profile (years)	Proportions with complications (%)									
			Post-herpetic neuralgia (months)				Ocular	Otic	Motor	Infections		≥1 overall
			NS	≥1	≥3	≥6				Skin	LRT	
Taiwan (Lin 2010) [136]	Hospitals (2.93% of 672783)	63% >40	52.5 (neurologic including PHN)				24.1	52.4		19.0	10.1	47.0
Australia (MacIntyre 2003) [102]	Hospitals (4718)	Mean 69	33.1 neurologic				16.0					58.5
Korea (Kim YM 2008) [11]	Dermatology (297)	NA	30.0				7.7	NA	1.4^a^	9.8		40.7
Korea (Lee 2006) [354]	Hospital (333)	NA	15.6				6.9	0.6	0.3^a^	1.5		NA
Korea (Park 2004) [26]	Dermatology (1089)	NA	7.4				3.2	0.2	0.4^a^	1.8		NA
Philippines (Jara 2010) [15]	Hospital (221)	Mean 43			2.3		5.0			6.3		12.2
Thailand (Aunhachoke 2011) [30]	Hospitals/tertiary centres (180)	Mean 59			19.4		7.2					
Singapore (Oh 1997) [340]	Hospital (67)	Mean 50	13.4				5.0			61.0		85.0
India (Abdul Latheef 2011) [12]	Hospital (205)	47% >40	10.2					1.0	0.5	13.6		34.6
Australia (Stein 2009) [104]	General Practice (379)^b^	100% >50	15.0				16.2^c^					61.3^c^
Japan (Akiyama 2000) [454]	Hospital (1432)	Mean 54							0.8			
India (Gopal 2010) [449]	Dermatology (100)								3.0			
Korea (Son 2011) [448]	Hospital (711)								2.1			
Korea (Min 2007) [418]	Dermatology (1787)	Mean 62						4.7				
Korea (Kim SH 2008) [372]	Dermatology (1496)						8.7					
New Zealand (Wallis 2014) [22]	General Practice (278)	55% >50					11.2					
New Zealand (Reid 2014) [17]	General Practice (339)	71% ≥51					6.3					
Thailand (Tunsuriyawong 2005) [154]	Hospital (399)	Mean 59	16.8				6.3					
Singapore (Goh 1997) [18]	Dermatology outpatients (164)	Mean 49		50.0	28.0	17.0						
Japan (Imafuku 2014) [359]	Dermatology (764)	Median 61		31.6	12.4	7.1						
Taiwan (Tsai TF 2014) [20]	General Practice & Hospitals (150)	Mean 65				20.7						
Korea (Song 2014) [25]	Specialist centres (151)	Mean 64			38.4	24.1						
Japan (Kurokawa 2002) [23]	Hospitals (263)	Mean 59				26.2						
Japan (Kurokawa 2007) [28]	Hospitals (316)				24.7	12.4						
Korea (Cho 2014) [357]	Dermatology (305)	Mean 53			6.2							
Taiwan (Jih 2009) [137]	National (34280)	33% >60		13.3	8.6							
Korea (Choi 2010) [130]	Cancer screening (282)			17.7								
China (Yang 2005) [355]	Dermatology (178)	NA		21.9								
Korea (Herr 2002) [356]	Dermatology (188)	NA		17.0								
China (Zhu 2009) [358]	Anaesthesiology (49)	Mean 65		20.4								
Korea (Cheong 2014) [96]	National (11502)	100% >50	20.6									
China (Song 2002) [21]	Dermatology (522)	Mean 47	6.9									
		Median (range)	17.0 (2.3–50.0)				7.2 (3.2–24.1)	1.0 (0.2–52.4)	0.8 (0.8–3.0)	10.0 (1.5–61.0)		47.0 (12.2–85.0)
		Quartile range (IQR)	11.3–23.0 (11.7)				6.3–11.2 (4.9)	0.6–4.7 (4.1)	0.5–1.8 (1.3)	5.6–15.0 (9.4)		37.7–59.9 (22.3)

*NS* Not specified, *LRT* Lower respiratory tract, *PHN* Post-herpetic neuralgia, *NA* Data not available, *IQR* Interquartile range

^a^Neurogenic bladder

^b^Incident cases

^c^Hospitalised patients

### Neuropathy, inflammation and secondary complications

HZ causes multifarious complications, especially inflammatory sequelae, which affect up to half or more of hospitalised HZ patients. The most common besides PHN involve the skin, eyes or, less often, ear, nose and throat (Table 5) [11, 12, 15, 26, 30, 102, 104, 136, 340, 354]. Uncommon complications include muscular weakness or paralysis and serious – sometimes life-threatening – cerebral or visceral inflammation. Elderly or immunocompromised individuals are more frequently and often worse affected [30, 96, 340, 370].

#### Eye

The most common and severe complications stem from VZV reactivation in trigeminal ganglion, which is the most frequently affected non-spinal location [11, 12, 23, 26, 29, 34, 371]. In particular, HZ ophthalmicus (HZO) arising from the ophthalmic division accounts for 5% to 25% of HZ cases in Asia-Pacific countries [11, 15, 17, 22, 30, 102, 104, 136, 154, 340, 354] (Table 5). The majority of cases, especially those accompanied by nose eruptions (Hutchinson's sign) [372–375], are complicated by secondary inflammation such as conjunctivitis, keratitis [376–378], scleritis, and uveitis/iridocyclitis [372–374, 379–381]. Such complications can cause glaucoma and are potentially blinding, especially if not treated promptly [379, 382]. However, inflammatory sequelae may not manifest until some time after an HZ episode [383]. Early and intensive antiviral therapy is particularly important for patients with HIV/AIDS, who tend to have worse and treatment-refractory inflammation, with resultant vision loss [384–386].

Molecular diagnostic techniques have revealed VZV reactivation to be a predominant cause of anterior uveitis in South and East Asian countries [387–389], with significantly increased risk within 1 year following HZ, especially HZO [390]. VZV is also a major cause of acute retinal necrosis [391–397] in the region, and of progressive outer retinal necrosis, which is usually seen in severely immunocompromised patients [398–402]. Both conditions have particularly poor visual outcomes, even when treated appropriately [375, 391, 392, 397, 399, 403–407].

Less common ocular complications include, corneal endotheliitis [408], retinochoroiditis [381], optic neuritis [409–411], oculomotor palsy [371], dacryoadenitis [412, 413], superior orbital fissure syndrome [414], orbital apex syndrome [415, 416], and central retinal vein occlusion [417].

#### Ear, nose and throat

Occasionally, VZV reactivation in the facial nerve genicular ganglion causes HZ oticus (Ramsay Hunt syndrome) [418, 419]. Patients often have multiple neuropathies and diverse symptoms, depending on the cranial and facial nerve branches involved. As elsewhere, the characteristic triad is facial palsy, auricular rash and ear pain [418, 420–422]; however, some cases affect the tongue, soft palate, or throat [423, 424], and the rash may precede facial paralysis, not appear until afterwards [420], or be absent [424]. Many patients experience hearing loss and vestibulo-cochlear symptoms such as tinnitus and vertigo [418, 420, 425, 426]. The seventh and eighth cranial nerves are most commonly affected [420, 427], but many atypical cranial neuropathies have also been identified [428–431]; Ramsay Hunt syndrome has been associated with dysphonia [46, 424, 429, 432, 433], laryngitis [434, 435], loss of taste [436], chronic cough [437], hiccups [435, 438], dysphagia [46, 424, 429, 439], and persistent vomiting [438]. Other rare complications include jugular foramen syndrome [440] and non-facial neuromotor deficits [441]. In addition to Ramsay Hunt syndrome, zoster sine herpete is increasingly recognised as a major aetiologic factor in idiopathic Bell’s palsy [442–444] and Ménière's disease [445, 446].

HZ oticus generally responds to prompt antiviral therapy, with good outcomes [421, 430]; however, half of patients may not recover fully from facial palsy [420, 421], with even lower rates among those who are older [427], have multiple cranial neuropathies [420], or are not treated [419]. The diversity of manifestations, often occurring sine herpete, creates potential for misdiagnosis [419]. It is vital for clinicians to remain alert to the possibility of HZ, so that treatment can begin early enough to avoid potentially life-threatening sequelae of VZV reactivation in the head and/or neck [438, 447].

#### Neuromuscular

Fewer than 5% of HZ cases in Asian patients cause motor neuropathy [448–450]. Though more common in the elderly or immunocompromised [450, 451], such complications also affect immunocompetent individuals [452, 453]. Among 711 Korean HZ patients, 2.1% had neuromotor weakness, predominantly affecting cranial rather than spinal nerves [448]. The incidence was 0.8% among 1432 Japanese patients [454] and 3% in an Indian cohort [449]. Such complications may arise before skin lesions appear and are likely underdiagnosed because they are masked by overriding pain [455, 456]; they may also occur some time after HZ onset [452, 457, 458]. Other presentations include gastrointestinal or bladder dysfunction [10], myelitis [450, 459–461], and myositis [462]. VZV infection is implicated in the aetiology of Guillain-Barré syndrome [463–465].

##### Face and eye

VZV reactivation is a common cause of Bell's palsy [466]. HZO may present as orbital myositis preceding skin eruptions [462], and sometimes causes complete ophthalmoplegia [467–472].

##### Limbs

Paresis of the ipsilateral shoulder or arm [473–477], occurs in 3–5% of HZ cases, but is underdiagnosed [478, 479]. Some cases are due to brachial plexopathy [480, 481], and others to Brown-Séquard hemiplegia [460, 482]. Lower limb neuropathy may manifest as foot drop [449, 483], and HZ paresis-induced femoral fracture has been reported [484].

##### Trunk and abdomen

Cervical myelitis can cause diaphragmatic paralysis with resultant dyspnoea [457, 485–487]. Segmental paresis may cause abdominal protrusion or pseudohernia [488–492], which are associated with gastrointestinal complications, notably constipation due to intestinal pseudo-obstruction or colonic ileus [488, 493–496].

##### Lumbosacral

Voiding dysfunction associated with sacral HZ is uncommon but not rare, especially among immunocompromised patients [497]. The incidence among 423 Taiwanese HZ patients was 4% overall, and 29% among those with lumbosacral HZ [498]. Urinary problems include neuropathic bladder [499], loss of voiding sensation [500] and occasional acute urinary retention [461, 501–503]. Other complications include faecal incontinence [504] rectal ulcer [505] and sciatica [506].

#### Vasculopathy

Though rare, vasculitic cerebral inflammation is the most serious neurologic complication of HZ; VZV from intracranial branches of the trigeminal nerve that invades and inflames the carotid artery or its branches, may cause fatal strokes [9, 10].

##### Cerebrovascular

Cerebral arteritis arising from HZ, especially HZO, significantly increases the risk of thromboembolic or haemorrhagic strokes [507, 508]. Those affected may have no visible lesions [509, 510] and strokes may be delayed [511–516] or occur without characteristic risk factors or symptoms [512, 513, 517, 518], leading HZ-associated stroke to be underdiagnosed [514, 519]. HIV/AIDS might be suspected in otherwise healthy younger individuals [518, 520].

##### Cerebral inflammation

Rare but severe and sometimes fatal neurologic complications include meningitis or meningo-encephalitis [521–526], cerebellitis [527], and encephalitis [528, 529]. Other reports include posterior reversible encephalopathy syndrome [530], CLIPPERS syndrome [531], and fatal meningo-encephalomyelitis [532]. Again, those affected are generally immunocompromised [9, 10], although accruing evidence suggests that such complications affect young and/or immunocompetent individuals more often than was supposed [521, 533–538].

##### Ocular

Cases of Horner’s syndrome and related cranial nerve palsies [539–542] have been attributed to HZO-induced arteritis and VZV meningitis [543]. Reported HZ retinopathies include visual loss due to central retinal artery occlusion and vasculitic chorioretinopathy [404, 544, 545].

##### Cardiovascular

Intriguing evidence hints at a link between HZ and cardiovascular disease in Asia. There are modestly elevated risks of acute coronary syndrome, arrhythmia and coronary artery disease following HZ [546, 547]. Herpetic infections may also be associated with atherosclerosis and thrombosis [546], with reports of HZ-associated deep vein thrombosis [548] and peripheral vascular disease [525]. Myocarditis [549] and unexplained recurrent asystole [550] following HZ have also been reported. Interestingly, however, Japanese patients with prior HZ had lower blood pressure than those with no history [551].

#### Skin

The most common dermatologic complications are secondary infections but numerous others have been described [9]. Rare complications include folliculitis, syringitis, and vasculitis [552–554]. HZ can also cause hair loss [555, 556].

##### Infections

Secondary infections complicate up to 30% of HZ cases [136], rising to 60% in vulnerable hospitalised patients [340]. The most common are staphylococcus or streptococcus superinfections [9, 10]. Other opportunistic organisms include moraxella [557], and aspergillus [558]. Bacterial infections are more likely in patients with diabetes [559, 560] and potentially serious, with necrotising fasciitis reported in one immunocompromised patient [561]. Immunocompromised patients, particularly those with HIV/AIDS, may rarely become reinfected by varicella despite a history of HZ [562].

##### Isotopic responses

HZ may render affected skin susceptible to other infections or dermatoses, for example, moloscum contagiosum, mycosis fungoides [154], erythema multiforme [563], and psoriasis [564, 565]. Diverse ‘isotopic’ responses at healed HZ sites include: vitiligo [566], fungal granuloma [567], furuncles [568], granulomatous reactions [569–574], erythemas [575–578], lichen planus [579, 580], morphea and bullae [581–583], perforating collagenosis [584, 585], keratolysis [586] and sarcoidosis [587, 588], reticulohystiocytosis [589], nodular degeneration [590, 591], verrucous hyperplasia [592], mucinosis [593], urticaria [594], prurigo nodularis [595], graft-versus-host reaction [596], drug-induced eruption [597], adenocarcinoma [598], leukaemia cutis [599], Kaposi’s sarcoma [600], and tufted angioma [601].

#### Dental

Jaw osteonecrosis and tooth loss are rare complications of trigeminal HZ. Cases have been reported in both immunocompetent and immunocompromised Asians; the mechanism remains unknown [602–610].

#### Disease associations

Emerging evidence from Taiwan and Japan suggests that patients with HZ are at increased subsequent risk of cardiovascular disease [546, 547], lymphoma [611] and other cancers [466, 612, 613], renal failure [614], SLE [615], multiple sclerosis [616], chronic fatigue syndrome [617], depression [618, 619] and erectile dysfunction [620]; however, the causality and significance of these associations remains unclear. Prevalent comorbidities such as metabolic syndrome disorders probably contribute to cardiovascular risk [547], while immunosuppression may underlie other conditions. Like HZ, kidney disease [160, 335], cancer [466, 612, 621, 622], and depression [158] are also associated with immunosuppression, and HZ may occur coincidentally either before or after these conditions manifest.

Anecdotal reports implicate VZV reactivation in rare conditions such as inappropriate secretion of antidiuretic hormone [85, 87, 623–626], drug-induced hypersensitivity syndrome [627], mononucleosis syndrome [628], thrombocytopenia [629], and graft-versus-host disease [630].

## Healthcare utilisation

As in the West, HZ-associated pain, morbidity and debility, result in heavy healthcare utilisation, especially among elderly patients, which imposes major burdens on healthcare systems and incurs substantial socio-economic costs in Asia-Pacific countries [20, 25, 30, 96, 102, 104, 136, 137, 140]. Although uncomplicated cases can usually be treated in community or outpatient settings, this usually entails several visits [20, 30, 96, 102]; Korean HZ patients averaged seven visits to a primary physician [96], and among 150 elderly Taiwanese patients, more than 80% consulted a doctor, with 20% hospitalised [20].

### Hospitalisation

Throughout Asia-Pacific, HZ is consistently among the disorders that dermatologists or pain specialists treat most often, and is the most common dermatosis in patients aged 60–70 years [149, 631–641]. Hospitalisation rates for HZ range from ~9–51/100000 PY (Table 6) [2, 7, 102, 104, 136–138, 140, 147]; complications account for around half of admissions (Table 5) [102, 104, 136] and, therefore, hospitalisation rates likewise increase with age and are highest among elderly or immunocompromised patients [96, 102, 104, 136, 138, 140, 147, 642]. Older patients and those with complications stay longer in hospital [136, 137].

**Table 6:** Herpes zoster hospitalisation rates, durations and associated healthcare costs in Asia-Pacific countries

Country (source)	Study period	Age profile (years)	Hospitalisations (100000 person years)	Hospitalisation duration (days)			Healthcare costs (US$ equivalent)^c^		
				Overall average	Complications		Per patient		Annual total (millions)
					None	With	Outpatient	Inpatient	
Taiwan (Lin 2010) [136]	2000–2005	All ages	14.6	8.3	8.0	8.6			7.7 in 2000 → 9.8 in 2004 (NT$ 319,000,000)
		≥80	105.1					204.0	
Taiwan (Jih 2009) [137]	2000–2006	All ages	16.1	8.3			78.4	1800.3	0.669773 (NT$ 14,147,543)
		0–20		6.06					
		≥60	59.5% of total	9.19					59.5% of total
Korea (Cheong 2014) [96]	2009	≥50 years	88.8^d^	10.93		14.75^e^	176.0 (161.0–240.0^f^)		63.2
Australia (Stein 2009) [104]	1998–2005	≥50 years	28.0^a^	6.8^a^	6.0	8.6 (5.2–14.6^b^)		3922.2^a^	27.5
		≥80	95.8						
Thailand (Aunhachoke 2011) [30]	2007–2008	Mean 59					90.4 (by 180 days follow-up)		~1.1% per capita income
Korea (Choi WS) [140]	2003–2007	All ages	22.0–32.0						75.9 increasing by 14–20% per year to 143.8
Australia (MacIntyre 2003) [102]	1998–1999	Mean 69	25.0	12.7					
		>50	53.0% of total						
		≥80	>150.0						
Korea (Song 2014) [25]	2009–2010	Mean 64		8.9					
Korea (Kim YJ) [138]	2011	All ages	51.2						
		≥80	266.3						
Australia (Carville 2010) [147]	2006–2007	All ages	9.1^a^						
		≥80	89.4^a^						
Australia (Araújo 2007) [7]	2000–2002	All ages	10.0^a^						
Australia (Heywood 2014) [2]	2006–2010	All ages	10.4						

^a^Principal diagnosis of herpes zoster

^b^Herpes zoster encephalitis

^c^Values not already stated in US$ equivalent, converted at average annual exchange rate in year prior to publication

^d^Based on *prevalence rate* of 18.54/1000 persons

^e^Post-herpetic neuralgia

^f^Severely immunocompromised

### Healthcare expenditure

Accordingly, HZ-related healthcare costs also increase proportionally with patients’ age and parallel rising incidence [136, 137]. Total expenditure in Taiwan rose by 1.22-fold from US$7.7 million in 2000 to $US 9.8 million in 2004 [136], and in Korea by 20% between 2003 and 2007 (Table 6) [140]. Direct healthcare costs in Thailand are equivalent to 1.1% of annual per capita income, a similar ratio to that in developed nations [30].

HZ in the Asia-Pacific region evidently imposes very substantial burdens on both patients and the wider community, strongly supporting the case for early intervention and prevention to reduce both HZ-related morbidity and associated healthcare expenditure [20, 25, 30, 96, 102, 104, 140].

## Management and prevention

Routine HZ management in Asia-Pacific is the same as elsewhere, mainly relying on antiviral and analgesic drugs to reduce the severity and duration of acute herpetic rash and pain, which may in turn decrease the risk and intensity of PHN [643, 644].

### Antiviral therapies

Standard care entails aciclovir, valaciclovir, or famciclovir for 7 days [643]. These are most effective if commenced within 3 days of onset [645] but as starting later may still be beneficial, patients with HZO, who are immunocompromised, have disseminated HZ, or are at high risk for PHN, should start antiviral therapy even beyond 72 hours [643–646]. Topical or oral aciclovir are effective in most mild-moderate HZ, including HZO [647–651]; nevertheless aciclovir is least favoured nowadays because its lower bioavailability necessitates more frequent dosing [9, 644, 652]. Though comparative data are sparse [643, 652], Asian studies support the use of other antivirals.

A meta-analysis including data from Taiwan [653, 654] and China, found valaciclovir and famciclovir superior to aciclovir in reducing HZ-associated pain, with comparable safety [652]. Valaciclovir resolves pain significantly faster than aciclovir [31, 653–655], while famciclovir has comparable efficacy but fewer adverse effects, and may also be more cost-effective [656, 657]. Penciclovir is also effective at lower doses than aciclovir, with better safety [658]. Specifically, aciclovir or its prodrug valaciclovir have been associated with neurotoxicity, and nephrotoxicity in East Asian patients with renal impairment [659–669], whereas famciclovir was safe and effective in patients with renal dysfunction [670, 671]. Treating HZ keratopathy with aciclovir ointment has been reported to cause superficial punctate keratopathy [672]. Ganciclovir may be a more effective alternative [673, 674], especially in aciclovir-resistant acute retinal necrosis [675]; success with intravitreal foscarnet following acyclovir failure has also been reported [676]. Valaciclovir and famciclovir are thought to have similar efficacy [643, 677], but Japanese researchers reported significantly faster pain relief with famciclovir [678]. In Caucasians, sirovudine treated acute HZ as safely and effectively as aciclovir but with fewer recurrences [679]; however, sirovudine has potentially fatal interactions with 5-fluorouracil prodrugs, which killed 18 Japanese cancer patients [680].

### Pain relief

Pain control is crucial to HZ management, not only for acute analgesia, but also because pain severity predicts PHN [9, 643, 644]. Although prompt antiviral therapy reduces acute HZ-associated pain, an updated meta-analysis concluded that aciclovir does not reduce the incidence of PHN and found insufficient evidence to evaluate the effect of other antiviral agents [681]. In practice, antiviral therapy is usually given concomitantly with corticosteroids or analgesics, according to the degree of pain.

#### Corticosteroids

Oral corticosteroids relieve pain, accelerate lesion healing and hasten functional recovery; however, being immunosuppressive they cannot be given without concomitant antiviral therapy. Moreover, corticosteroids should be used with caution in patients with common comorbidities such as diabetes and hypertension and do not prevent or relieve PHN [643, 645, 646]. Nevertheless, corticosteroid injections have successfully treated cases of Ramsay Hunt syndrome and refractory PHN in Chinese patients [682, 683].

#### Analgesia

Pain relief should start early and be intensified as necessary during acute HZ to control pain and reduce the likelihood of developing PHN, which is much harder to treat [656, 684, 685]. Pain management steps-up from first-line acetaminophen or non-steroidal anti-inflammatory drugs to opioid narcotics for moderate-to-severe pain, to which anticonvulsants, tricyclic antidepressants or corticosteroids are added if pain remains uncontrolled [646]. The same second- and third-line analgesics are used to treat PHN if topical agents prove ineffective [643, 646].

Indian physicians have reported rapid and effective pain relief with topical acetylsalicylic acid dissolved in chloroform [686]. Others confirmed the efficacy of the anticonvulsants pregabalin and gabapentin for relieving acute herpetic pain [687, 688], and found pregabalin superior to amitriptyline for PHN [684]. Compared with placebo, pregabalin relieved PHN and was also associated with decreased sleep interference and significant improvements in health-related QoL [689]. Gabapentin is another effective and well-tolerated treatment for PHN [690] and combined with morphine in another trial, reduced pain more than either agent alone [691]. Chinese investigators found oxycodone-acetaminophen effective, safe and superior to other analgesics in HZ and PHN [692–696]. Reports from Australia, Japan, New Zealand and Taiwan have affirmed the efficacy of topical [697–699] or injected lidocaine [700, 701]. Transdermal fentanyl was found to be more effective than tramadol, providing excellent pain relief in HZ and PHN and improving QoL, both alone [702, 703] and combined with clodine [704]. Other experimental therapies with good reported outcomes in patients from Asia-Pacific countries include: topical interferon alpha [705, 706], adenine arabinoside (vidarabine) [707, 708], povidone-iodine [709], transdermal ribavirin [710], bromovinyl deoxyuridine (brivudin) [711], intravenous prostaglandin E1 [355, 712–715], the anticoagulant argatroban [716]; the tricyclic antidepressant milnacipran [717], human immunoglobulin [718], injected methylcobalamin [719], and botulinum toxin [720].

Nerve blockade is increasingly popular for treating intractable PHN or preventing PHN by reducing severe pain, particularly in East Asia [363, 721–742]; ambulatory patient-controlled systems have been successful in Taiwan [743, 744]. However, the true efficacy of nerve blocks is difficult to quantify because many trials were uncontrolled [9]. Furthermore, epidural injections require utmost caution due to the potential for infection and/or life-threatening complications [745–755]. An implanted injection port may lessen the risk of infection [756].

Neurosurgery may trigger HZ [269–271] and though neuroablation has also been used, its efficacy is unproven and it may exacerbate PHN [757]; neuromodulation may provide an alternative but needs further investigation [758, 759].

### Alternative treatment modalities

Asia-Pacific authors have contributed substantial literature on alternative HZ therapies (Additional file 1). Success has been reported with herbal decoctions, acupuncture and other traditional Chinese medicine techniques, energy-based modalities, and combination therapies. Treatments evaluated in intractable HZ and PHN, include *Ganoderma lucidum* [760, 761], Keishikajutsubuto and Bushi-matsu [762], bee venom [763], intravenous vitamin C [764], computed tomography-guided radiofrequency thermo-coagulation [765], spinal cord stimulation [758], ultrasound-guided pulsed radiofrequency [766], and scrambler therapy [767]. Although many studies found these alternatives superior to conventional medicine, the strength of this evidence is dubious – due to conceptual differences between traditional Asian and Western medicine, many such studies do not conform to conventional evidence-based precepts [768–770].

### Unmet treatment needs

Despite an armamentarium of potentially effective medications, current therapies have limitations and are not used to best advantage in older patients, who bear the brunt of HZ [646]. In particular, many patients start therapy too late, the optimal combination of therapies remains uncertain, antiviral agents are underprescribed [22, 645, 771], and even with prompt intervention a substantial proportion remain refractory to treatment; up to 20% of patients with PHN still have persistent neuralgia after 6 months (Table 5) [18–20, 23, 28, 359, 771]. Aciclovir-resistant ocular VZV infections have been encountered [675, 676, 772, 773].

Given the substantial and rising burden of HZ and formidable treatment challenges facing physicians in the Asia-Pacific region, especially among elderly, immunocompromised or other high risk adults, such as those with renal disease, prevention is both rational and appealing [20, 25, 94–96, 104, 136, 140, 144–147, 160, 644]. Accordingly, investigators have evaluated HZ prophylaxis with antiviral drugs or vaccines.

### Prevention approaches

#### Antiviral prophylaxis in high-risk patients

HZ is a very common complication following HSCT [170, 774]. Long-term prophylaxis with low-dose aciclovir [775–778] or valaciclovir [779] significantly reduces the incidence of VZV reactivation and serious complications, and is recommended for the duration of immunotherapy and continuing through 1 year after HSCT [776, 778]. Such prophylaxis also prevents HZ in patients receiving bortezomib [780, 781]. Similarly, no bone marrow transplant recipients developed HZ during 3–6 months of low-dose aciclovir and ganciclovir therapy; however, rapid onset of HZ after antiviral therapy was discontinued highlighted the need for ongoing prophylaxis [782]. Immunisation against VZV may be necessary to preclude HZ in high-risk patients [776]; therefore, Asian researchers have evaluated the potential of HZ prophylaxis with varicella vaccines.

#### VZV immunisation

A survey of Japanese paediatricians, who might have enhanced immunity through VZV re-exposures, confirmed lower incidence of HZ than the general population, suggesting that varicella vaccine may likewise protect against HZ [319]. Also in Japan, immunisation with live varicella vaccine (Oka/BIKEN) enhanced CMI to VZV in adults age ≥50 years [783, 784], and in subjects aged 60–70 with or without diabetes [785]. A case of recurrent HZO cured by varicella vaccination was also reported [786]. In studies that evaluated live-attenuated VZV vaccine in HSCT [774, 787], Oka/RIT (Varilrix™ Glaxo-SmithKline Biologicals, Rixensart, Belgium) was safe but poorly immunogenic. A systematic review concluded that although inactivated VZV vaccine may reduce HZ severity in stem cell transplant recipients, more research was required [788]. Thus, alternative approaches to preventing HZ in such patients are probably needed [787].

During a 20-year endeavour to develop the first specific HZ vaccine, researchers reformulated Oka/Merck varicella vaccine (Varivax® Merck & Co. Inc., Whitehouse Station, NJ, USA.) to produce a higher-titre live-attenuated VZV vaccine – Zostavax® (Merck & Co. Inc.) [10, 789]. In the culminating Shingles Prevention Study (SPS), Zostavax® immunisation reduced the incidence of HZ among adults ≥60 years old by 51%, PHN by 67% and the HZ-related burden of illness by 61%, as well as improving QoL and performance of daily activities in subjects who developed HZ [95]. The US Food and Drug Administration licenced Zostavax® in 2006 [644]; it was also approved by the European Medicines Agency [96] and subsequently by authorities in Australia [644] New Zealand [652], Korea [790, 791], Malaysia [792], and other Asian countries [793–796]. The US Centers for Disease Control and Prevention Advisory Committee on Immunization Practices (ACIP), recommends routine vaccination for all non-contraindicated persons age ≥60 years; importantly though, Zostavax® is not indicated to treat extant HZ or PHN [10]. An independent review of SPS concluded that there is insufficient proof that Zostavax® prevents PHN beyond reducing the incidence of HZ. Further, since SPS patients were predominantly Caucasian, its findings might not apply to other races [797].

##### Zostavax® in Asia-Pacific

Production shortfalls of Zostavax® after initial licensure restricted its supply [1, 2]. Though since resolved, this issue affected vaccine availability and uptake in Asia-Pacific countries [2]. For example, 2011 vaccination rates among Australian inpatients in 2011 were 34% for HZ, compared with 52% for pneumococcal vaccine and 64% for influenza [798]. This explains the scarcity of Asian data on Zostavax®. One pilot study of 21 healthy adults ≥30 years old, including eight Filipinos, found Zostavax® to be immunogenic and generally well-tolerated [799].

##### Cost-effectiveness

The substantial economic burden of HZ includes direct healthcare costs as well as indirect costs of disability and lost productivity [10, 30, 140]. US and European researchers have modelled the pharmaco-economics of HZ vaccination. US estimates of cost per quality-adjusted life-years gained (QALY), varied widely depending on the assumptions used. Routine immunisation at age 70 or 60 cost US$ 37000 and US$ 86000 respectively, within the range of standard thresholds, whereas the estimated cost of US$ 287000 at age 50 was deemed too high [800]. Analyses in European countries predicted that vaccinating adults above the ages of 50, 60, 65 or aged 70–79 would be cost-effective, with high likelihood of not exceeding accepted thresholds of £ 30000 in the United Kingdom or € 30000 in Belgium [801]. A systematic review of 11 US and European studies concluded that vaccination at age 65–70 would probably be cost-effective in terms of QALYs gained, assuming it confers more than 10–15 years’ protection against PHN [802]; however, cost-effectiveness data from high-income countries may not apply worldwide, because healthcare provision and costs differ considerably between nations [801, 802]. Although high and rising healthcare costs of treating HZ documented in several Asia-Pacific countries [30, 104, 136, 137, 140] suggest that it would probably be no less cost-effective to immunise elders from this region than others, specific pharmacoeconomic data are lacking [790, 802]; such analyses are needed urgently to resolve current uncertainties and inform evidence-based decision-making by healthcare funders and providers.

## HZ immunisation guidelines and recommendations

Given the potential for HZ immunisation to efficaciously and safely reduce the associated burden of illness [10], as well as cost-effectiveness considerations, organisations in several Asia-Pacific countries have incorporated HZ vaccination into adult immunisation schedules (Table 7) [2, 5, 8, 642, 791–796]. Neither Japan nor Singapore has such guidelines and The Association of Physicians of India does not recommend HZ immunisation, due to lack of national epidemiology data [803].

**Table 7:** Asia-Pacific guidelines for immunisation against herpes zoster

Country	Title	Issuing organisation		Year (last update)	Age thresholds	
		Name	Status		Routine	Optional
Australia [8]	The Australian Immunisation Handbook	Technical Advisory Group on Immunisation	Government	2015	60–79	
Australia & New Zealand [5]	Immunisation of Older People	Australia & New Zealand Society for Geriatric Medicine	Society	2011	≥60	
India [803]	Medicine Update. Adult Immunization	Association of Physicians of India	Society	2013	No recommendation^a^	
Indonesia [793]	Adult Immunization Schedule	Specialist Doctors Association of Indonesia	Society	2014	≥50	
Malaysia [792]	Position Statement on Vaccination Against the Herpes Zoster Virus in Older Adults	Malaysian Society of Geriatric Medicine	Society	2014	60–79	50–59
New Zealand [642]	Immunisation Handbook	New Zealand Ministry of Health	Government	2016	>50	
Philippines [794]	Handbook on Adult Immunization for Filipinos	Philippine Society for Microbiology and Infectious Diseases	Society	2012	≥60	
South Korea [791]	Recommended Immunization Schedule for Adults in Korea	Korean Society of Infectious Diseases	Society	2012	>65	50–59
Taiwan [796]	Clinical Handbook for Adult Immunization	Taiwan Association of Family Medicine	Society	2010	≥60	
Thailand [795]	Recommended Adult and Elderly Immunization Schedule	Thailand Royal College of Physicians	Society	2014	≥60	

^a^The Association of Physicians of India does not recommend herpes zoster vaccine for adults, due to lack of reliable data on the epidemiology and burden of herpes zoster in India

Most HZ immunisation guidelines are published by professional societies, with only Australia and New Zealand having government-issued guidelines [8, 642]. Since Asian epidemiological and clinical data are sparse, local guidelines generally follow the ACIP [10], with very similar recommendations for indicated and non-licenced uses, administration, and precautions or contraindications (Table 8). Like ACIP, most Asia-Pacific guidelines recommend routine immunisation from age 60; however, some age thresholds vary. The New Zealand Ministry of Health and Indonesia Specialist Doctors Association both recommend immunisation from age 50 [642, 793], though this is not state funded. The Korean Society of Infectious Diseases recommends routine vaccination above age 65 [791], with vaccination from age 50 at patients’ own discretion. Similarly, The Australian Department of Health [8] and the Malaysian Society of Geriatric Medicine [792] do not recommend routine vaccination for persons aged 50–59, but sanction this as a personal option, with the caveats that the duration of protection remains undetermined. However, the Malaysian Society of Geriatric Medicine does not recommend vaccinating patients older than 80, due to lack of efficacy, and the Australian guidelines likewise note that vaccination may confer less clinical benefit in this age group [8]. The New Zealand guidelines [642] uniquely specify active untreated tuberculosis as a contraindication and the Australian guidelines are alone in indicating vaccination of persons from age 50 who are household contacts of immunocompromised individuals [8]. Based on evidence that co-administering Zostavax® with pneumococcal polysaccharide vaccine may reduce its immunogenicity compared with administration 4 weeks apart [804], the Australian and New Zealand Society for Geriatric Medicine recommended against giving these vaccines concomitantly [5]; however, a later study suggested that this may not compromise Zostavax® effectiveness [8, 805], and the Australian 2015 guidelines recommend that Zostavax® can be given at the same time as pneumococcal polysaccharide vaccine, using separate syringes and injection sites [8].

**Table 8:** Concordance between herpes zoster immunisation recommendations from US ACIP and Asia-Pacific countries

Indications and administration	• Routine immunisation with one dose of HZ vaccine for all persons age ≥60: – With or without prior HZ – With chronic medical conditions (eg, chronic kidney disease, diabetes, rheumatoid arthritis, lung disease), except those listed as contraindications or precautions • HZ vaccine can be co-administered with other indicated adult vaccines, eg, influenza
Unlicensed categories/ purposes	• Persons immunised with varicella zoster vaccine • Persons younger than the minimum recommended or optional age threshold • HZ vaccine is not to be used to treat existing HZ or its complications
Precautions	• Moderate/severe acute illness • Anticipated immunosuppression • Anti-herpetic pharmacotherapy
Contraindications	• Hypersensitivity to vaccine components • Morbid or medical immunosuppression or immunodeficiency: – HIV/AIDS – Transplant recipients – Systemic immunosuppressive therapy (including high-dose steroids and recombinant immune modulators • Pregnancy

*HZ* Herpes zoster, *US ACIP* United States Centers for Disease Control and Prevention Advisory Committee on Immunization Practices, *HIV/AIDS* human immunodeficiency virus/acquired immunodeficiency syndrome

## Discussion

Ours is the most comprehensive audit yet of HZ in the Asia-Pacific region. We have reviewed almost every available paper published on the subject over 21 years since 1994 in 14 countries whose populations constitute the majority of not only Asians, but indeed the global population. The information collated provides a valuable resource and reference by which to gauge future progress.

### Knowledge and data gaps

Contrary to a preconception that there may be a dearth of Asian data, we discovered a wealth of evidence on all aspects of HZ. However, our review does affirm that Asian data are patchy; in particular, there is very little information from South-East Asian populations numbering hundreds of millions, which probably reflects pragmatic healthcare imperatives in resource-limited settings [3]. Limiting the scope of the survey to 21 years may have missed epidemiology data published prior to 1994. The review may have excluded some relevant articles which did not specify that VZV infection was reactivated. It is also possible that some articles with no country affiliation field in the databases searched may have been overlooked; however, searching several databases reduced this possibility. Epidemiology data from Indonesia, Malaysia, the Philippines, and Vietnam are needed to provide a more balanced picture. There are also limited Asian data on the safety and efficacy of the HZ vaccine and, in particular, its cost-effectiveness [137, 140, 790, 802].

## Conclusions

Data gaps notwithstanding, there is compelling evidence to conclude that the epidemiology and risk factors for HZ in the Asia-Pacific region are not remarkably different from those in Western populations [7]. However, with a vastly larger absolute aged population, Asia bears a unique burden of HZ. The estimated number of Asians^1^ age >60 in 2015 is 489,397,421, which by 2035 will nearly double to 924,520,454 [806]. Assuming an annual incidence of HZ in unvaccinated individuals of approximately 12/1000 person-years [136, 137], this equates to approximately 16089 new HZ cases daily in 2015 and 30395 in 2035, more than half of which might be prevented by HZ immunisation. Data affirming rising incidence of HZ in countries across Asia-Pacific bear out these projections. Consequently, HZ in the Asia-Pacific region exacts huge and increasing tolls of morbidity, debility and diminished life quality that incur significant healthcare expenditure and indirect socioeconomic costs [20, 25, 30, 96, 104, 136, 137, 140].

This review also highlights that HZ is uniquely complex among infectious diseases, often complicated and defies disciplinary boundaries. Despite a plethora of conventional and alternative treatments, none is singularly effective [25]; current approaches can go only so far to alleviating the disease burden, especially among the elderly who constitute most patients, and then at considerable expense [95]. Thus, the rationale for prevention is very strong [20, 102, 145]. Yet although a specific HZ vaccine is available and recommended by immunisation guidelines in many countries, it remains underused. This typifies how preventive healthcare for the elderly continues to be neglected in Asia, despite repeated calls to make this a higher public health priority [3, 4, 6]. It is more urgent now than ever to address this situation.

### Call to action

We look forward to being able to report in future that advances in preventive healthcare have alleviated the growing burden of HZ in the Asia-Pacific region. This audit is just a beginning – realising this ambition will be impossible without redoubled efforts by the medical fraternity, healthcare authorities and all other stakeholders to change prevailing mindsets and afford higher priority to adult immunisation in general and HZ in particular. We urge all concerned to heed this call to action.

## Additional file

Additional file 1: Appendix 1. Alternative approaches to treating herpes zoster and herpetic neuralgia. (PDF 340 kb)

## Abbreviations

ACIP: Advisory Committee on Immunization Practices
AIDS: Acquired immunodeficiency syndrome
CLIPPERS: Chronic lymphocytic inflammation with pontine perivascular enhancement responsive to steroids
CMI: Cell-mediated immunity
HAART: Highly-active antiretroviral therapy
HIV: Human immunodeficiency virus
HSCT: Haematopoietic stem cell transplant
HZ: Herpes zoster
HZO: Herpes zoster ophthalmicus
PHN: Post-herpetic neuralgia
PY: Person-years
QoL: Quality of life
SLE: Systemic lupus erythematosus
SPS: Shingles Prevention Study
US: United States
VZV: Varicella zoster virus
WHO: World Health Organization

## Footnotes

1 Afghanistan, Bangladesh, Bhutan, Brunei, Burma, Cambodia, China, Hong Kong, India, Indonesia, Iran, Japan, Laos, Macau, Malaysia, Maldives, Mongolia, Nepal, North Korea, Pakistan, Philippines, Singapore, South Korea, Sri Lanka, Taiwan, Thailand, Timor-Leste, Vietnam.
