# Supplementary material for: Looking back to move forward: a twenty-year audit of herpes zoster in Asia-Pacific
Source: BMC Infect Dis. 2017 Mar 15;17:213. doi: 10.1186/s12879-017-2198-y (PMC5353949; doi:10.1186/s12879-017-2198-y)
Supplement: Additional file 1: Appendix 1. — Alternative approaches to treating herpes zoster and herpetic neuralgia. (PDF 340 kb) [file 12879_2017_2198_MOESM1_ESM.pdf]

Appendix. Alternative approaches to treating herpes zoster and herpetic neuralgia

Modality	Effector	Study method (Number of HZ patients)	Reported efficacy of evaluated effector against HZ and/or herpetic neuralgia	Country[a] (Source)
**Botanic**	*Undaria pinnatifida*	Effect of extract of *U. pinnatifida* (GFS) in 21 patients with herpetic infections (n=2)	GFS inhibited herpes virus reactivation and increased healing rate of herpetic outbreaks	Australia (Cooper 2002)[1]
	TCM herbs (unspecified decoction)	Randomised to TCM vs. western medicine: 1994–1995 (NR)	TCM better than western medicine (TC 93.3% vs. 85.8%, effective 75.5% vs. 52.2%)	China (Chen 2007)[2]
	Ganoderma lucidum	Effect of *G. lucidum* on acute HZ pain and PHN (n=4)	Decreased severe HZ pain and intractable PHN	Japan (Hijikata 1998)[3]
		Effect of formula containing *G. lucidum* on acute HZ pain (n=5)	Rapid pain relief in a few days, almost complete by 10 days, no PHN through 1 year follow-up	Japan (Hijikata 2005)[4]
	Keishikajutsubuto & Bushi-matsu	Effect of two herbs in PHN aggravated by cold environment (n=15)	Treatment is a promising way to treat intractable PHN	Japan (Nakanishi 2012)[5]
	Keisi-bushi-to & Hachimi-jio-gan	Effect of oriental herbal medicines on HZ pain (n=8)	Pain symptoms relieved in 2/3 treated with Keisi-bushi-to and 4/5 with Hachimi-jio-gan granules	Japan (Aki 2002)[6]
	Yokukansan	Effect of Yokukansan in HZ pain and PHN (NR)	Yokukansan more effective than conventional analgesics	Japan (Nakamura 2009)[7]
	Sairei-to	Pain relief in HZ (NR)	Analgesic efficacy 76.8%	Japan (Shukuwa 2002)[8]
	Clinacanthus nutans	Systematic review and meta-analysis of 4 RCTs (n=126)	Relative risk of 3.2 for 3-day full crusting (IQR 0.97–10.58), suggests beneficial effect in HZ	Thailand (Kongkaew 2011)[9]
		Randomised to *C. nutans* 5% extract vs. placebo (n=51)	Faster healing and pain reduction vs. placebo (P<0.01), with no side effects.	Thailand (Sangkitporn 1995)[10]
		Randomised to *C. nutans* 5% cream vs. placebo (n=75)	Faster lesion healing (27.5% vs. 8.5%)	Thailand (Charuwichitratana 1996)[11]
	Radix isatidis	*R. isatidis* prophylaxis against HZ in multiple myeloma treated with bortezomib (n=30)	Higher incidence of VZV reactivation without prophylaxis (25.0% vs. 0.0%, P=0.003)	China (Guo 2011)[12]
**Molecular**	Hyperbaric oxygen (HBO_2)	Randomised to HBO_2 + medication vs. medication (n=68)	HBO_2 enhanced efficacy (97.2% vs. 81.3%, P<0.05), faster healing, lower PHN rate and less depression	China (Peng 2012)[13]
	Vitamin C	Effect of IV vitamin C in refractory HZ (n=1)	Treatment immediately reduced pain	Korea (Byun 2011)[14]
	Bee venom	Effect of bee venom in refractory PHN (n=1)	Self-reported pain score fell from 8/10 to 2/10, with no adverse effects or rebound through 1 year follow-up	Korea (Lee 2014)[15]
**Mechanic**	Intramuscular stimulation (IMS)	Effect of IMS in treating PHN (n=4)	Self-reported pain decreased by more than half	Korea (Jung 2010)[16]
	Acupuncture (AP)	Meta-analysis of 10 RCTs (NR)	Superior clinical cure and analgesia to western medicine (P<0.01), more high-quality studies needed	China (Yu 2007)[17]
		Randomised to AP vs. ibuprofen + famotidine + IV vitamins B_1 & B_{12} (n=24)	Treatment superior (TE 91.6% vs. 83.3%)	China (Fang 2011)[18]
		Case series of nerve cell puncturing (n=48)	Good therapeutic results	China (Ni 2008)[19]
	Cupping	Meta-analysis of 135 RCTs:[b] 1992–2010 (NR)	Potential effect, but need more well designed trials	Australia (Cao 2012)[20]
		Systematic review of 73 RCTs, 22 trials, 373 case series, 82 cases: 1959–2008 (NR)	Potential benefit but more rigorously designed trials warranted	China (Cao 2010)[21]

	Blood letting	Systematic review of 98,526 cases involving 261 diseases (NR)	Bleeding therapy has evident advantages for treating HZ	China (Chen 2009)[22]
		Case study of bleeding point ear apex (n=1)	Immediate relief of most pain, two treatments cured	China (Wang 2006)[23]
**Energetic**	Far-infrared radiation	Effect of far-infrared radiation in HZ (n=50)	Superior healing and pain relief vs. control	China (Zhang 1994)[24]
	CT-guided radiofrequency (RF) thermocoagulation	Effect of CT-guided RF dorsal root ganglion thermocoagulation in intractable PHN (n=16)	Hyperalgesia disappeared, immediate self-reported pain scores fell from 7–9/10 to 2–3/10, with no relapse	China (Huang 2008)[25]
	Laser	Effect of combi laser therapy in PHN (n=50)	Cure 88.0% after 20 exposures, relief 10% included four HZ ophthalmicus cases	India (Mittal 1996)[26]
		Case-control study of adding He-Ne laser to medication in HZ keratitis (NR)	Laser enhanced efficacy with accelerated healing and better pain relief (P<0.05)	China (Li 2012)[27]
	Temporary electric spinal cord stimulation (SCS)	Effect of adding SCS to epidural block in refractory PHN (n=14)	SCS reduced self-reported pain and spared epidural anaesthesia	Japan (Moriyama 2009)[28]
	Light-emitting diode (LED)	Randomised to LED + IV acyclovir + analgesia vs. IV acyclovir + analgesia (n=60)	LED accelerated relief of acute pain (1.4 vs. 1.8 weeks, P<0.05) but had no effect on onset of PHN	Korea (Jung 2013)[29]
		HZ ophthalmicus patients randomised to 830 nm LED vs. oral famciclovir (n=28)	LED accelerated healing (13.1 days vs. 15.9 days, P=0.006) and reduced self-reported pain from day 4	Korea (Park 2013)[30]
	Ultrasound (US)-guided pulsed RF	Effect of US-guided pulsed RF in refractory facial HZ (n=1)	Pain reduced, persisting through 6-months follow-up, with no need for further management	Korea (Lim 2013)[31]
	Scrambler	Effect of scrambler therapy in refractory PHN (n=3)	Self-reported pain decreased by 50% after 10 sessions	Korea (Ko 2013)[32]
**Combined**	AP + blood-letting with cupping & surround needling	Randomised to combination vs. oral + topical aciclovir (n=240)	Combination more effective (92.5% vs. 55.8%, P<0.01), with more rapid pain relief and healing	China (Huo. 2007)[33]
	Acupoint selection + AP + cupping	Randomised to combination at differential acupoints vs. affected areas (n=86)	Targeted-therapy superior (cure & effective 93.0% vs. 67.4%, P<0.01), lower PHN rate (2.3% vs. 14.0%)	China (Pan 2011)[34]
	AP + electro-AP, moxibustion hot needle, tapping + cupping	Randomised to various mechanic TCM modalities vs. western medicine (n=500)	No significant difference in efficacy, but TCM more cost-effective	China (Li 2012)[35]
	Electro AP + AP or cotton moxibustion or hot needle or tapping/cupping	Randomised to various TCM modalities vs. oral valaciclovir + vitamin B_1 (n=189)	No significant differences in clinical cure efficacy but TCM significantly reduced pain from post-treatment day 7 (P<0.05)	China (Huang 2012)[36]
	Collateral-pricking + 'healthy energy strengthening'	Retrospective comparison of collateral pricking vs. aciclovir (n=66)	Treatment superior (cure 95.6% vs. 75.6%, P<0.05)	China (Yu 2005)[37]
	Needling + moxibustion	Randomised to moxibustion at area of surround needling vs. needling only (n=72)	Treatment more effective (97.4% vs. 85.3%, P<0.05), faster healing and reduced residual neuralgia	China (Zhang 2007)[38]
	AP + point injection	Effect of combination in PHN (n=30)	Combination has curative effect on PHN (TE 90%)	China (Wang 2008)[39]
	Needling + thin-cotton moxibustion	Randomised to combination vs. oral + topical aciclovir (n=80)	Treatment superior (effective 78.6% vs. 39.5%, P<0.05)	China (Tian 2011)[40]
	Plum-blossom needle tapping + moxibustion	Randomised to combination vs. topical aciclovir + oral valaciclovir + vitamin B_{12} (n=120)	Treatment superior (cure 80.0% vs. 45.0%, P<0.10; TE 98.3% vs. 71.7%, P<0.05) and faster, recurrence 1.6% vs. 13.3%	China (Yang 2012)[41]
	Percussopunctato + cupping	Effect of combination in HZ and PHN (n=33)	TE 100%: cure 60.6%, good effect 33.3%, effect 6%	China (Han 2003)[42]

	Ear tapping & pressing + acupoint vitamin B_{12} injection	Randomised to combination vs. standard western medicine (n=116)	Cure 60% vs. 28.6%, effective 83.3% vs. 50.0%, (P<0.05), faster pain relief and cessation (P<0.01)	China (Wu 2007)[43]
	Blood-letting & cupping + UV irradiation	Randomised to combination vs. standard therapy including aciclovir (n=130)	Higher and faster cure & effective rates (respectively, 76.9% vs. 38.5% & 90.8% vs. 66.2%, P<0.01), lower PHN rate (3.1% vs. 12.3%, P<0.05)	China (Ouyang 2009)[44]
	Electro-AP + needling	Randomised to combination vs. valaciclovir + vitamin B_1 (n=80)	Combination superior (cure 75.0% vs. 37.5%, effective 92.5% vs. 67.5, with faster healing (P<0.01)	China (Li 2009)[45]
	Electro-AP + blood-letting & cupping	Randomised to combination vs. valaciclovir + indomethacin + vitamins B_1 & B_{12} (n=53)	Cure & effective 96.8% vs. 81.8% (P<0.05)	China (Liu 2009)[46]
	Electro-AP + collateral pricking & cupping	Randomised to combination vs. oral valaciclovir + indomethacin (n=53)	Combination superior (cure & effective 93.5% vs. 72.7%, P<0.01)	China (Liu 2013)[47]
	AP & cupping + blood letting	Case report (n=1)	One treatment reduced pain and five cured HZ, with no residual pain or sequelae at 3 months	China (Hu 2001)[48]
	Electronic meridian detection + blood-letting & cupping	Randomised to combination vs. oral acyclovir (n=200)	Treatment superior (TE 100% vs. 60%, P<0.001), faster pain relief and healing, PHN rate 0.0% vs. 26%	China (Dai 2011)[49]
	Variable frequency electro-AP + herbal moxibustion	Randomised to combination vs. ibuprofen + vitamins B_1 & E (n=37)	Treatment superior to medication (effective 94.44% vs. 73.68%, P<0.05)	China (Wang 2012)[50]
	Swift needling + medication	Randomised to fire-AP + oral valaciclovir + vitamin B_1 vs. oral valaciclovir + vitamin B_1 (n=79)	Combination superior (TE 95.1% vs. 89.5%, P<0.05)	China (Fan 2013)[51]
	Subcutaneous needling + laser	PHN patients randomised to electro-AP + laser vs. electro-AP (n=102)	Combination superior (TE 94.1% vs. 68.6%, recovery 56.9% vs. 35.3%, P<0.05), with faster action and cure and less relapse (6.9% vs. 44.4%, P<0.05)	China (Guo 2014)[52]
	Encircled AP + valaciclovir	Effect of encircled AP + oral valaciclovir vs. oral valaciclovir (n=60)	Combination superior, faster healing and pain relief	China (He 2007)[53]
	Surround AP + He-Ne laser	Randomised to surround AP + IV aciclovir + He-Ne laser vs. IV & topical aciclovir (n=60)	Combination superior in effective rate, healing, pain relief and time to cure (P<0.05)	China (Song 2013)[554]
	TCM + mecobalamin	PHN patients randomised to mecobalamin or TCM vs, mecobalamin + TCM (n=160)	Combination superior (effective 90.9% vs. 72% mecobalamin & 70.9% TCM, P<0.01)	China (Tian 2005)[55]
	Linear polarised near-infrared light (LPIR) + fentanyl patch	Randomised to combination vs. LPIR or fentanyl patch (n=30)	Combination superior (80% vs. 60% & 70%, P<0.05), self-reported pain fell from baseline (P<0.05)	China (Wang 2007)[56]
	Electro-AP + herbal medicine	Randomised to combination vs. oral + topical aciclovir + mecobalamin (n=62)	Combination superior (P<0.01)	China (Wang 2012)[57]
	Oral TCM + AP, moxibustion, corticosteroids, antivirals, analgesia and trophic nerve	Randomised to combination vs. conventional antiviral and analgesic agents ± corticosteroids ± AP & moxibustion (n=128)	Composite treatment with oral TCM significantly eased self-reported pain and improved quality of life	China (Xie 2003)[58]

AP acupuncture, *HZ* herpes zoster, *NR* not reported, *UV* ultraviolet, *PHN* post-herpetic neuralgia, *HBO₂* hyperbaric oxygen, *RCT* randomised controlled trial, *TCM* traditional Chinese medicine, *LED* light-emitting diode, *IV* intravenous, *US* ultrasound, *IQR* interquartile range, *TC* total cure, *TE* total effective, *RF* radiofrequency, *CT* computed tomography, *SCS* spinal cord stimulation.

[a] Main affiliation of first author.

[b] Included cupping types: wet, retained, moving, flash, medicinal, needle, combined.

References

1. Cooper R, Dragar C, Elliot K, Fitton JH, Godwin J, Thompson K. GFS, a preparation of Tasmanian Undaria pinnatifida is associated with healing and inhibition of reactivation of Herpes. BMC Complement Altern Med. 2002;2:11.
2. Chen HM. Zoster Chinese medicine treatment. Zhonghua Shi Yan He Lin Chuang Bing Du Xue Za Zhi. 2007;21:285-7.
3. Hijikata Y, Yamada S. Effect of Ganoderma lucidum on postherpetic neuralgia. Am J Chin Med. 1998;26:375-81.
4. Hijikata Y, Yasuhara A, Sahashi Y. Effect of an herbal formula containing Ganoderma lucidum on reduction of herpes zoster pain: a pilot clinical trial. Am J Chin Med. 2005;33:517-23.
5. Nakanishi M, Arimitsu J, Kageyama M, Otsuka S, Inoue T, Nishida S, et al. Efficacy of traditional Japanese herbal medicines-Keishikajutsubuto (TJ-18) and Bushi-matsu (TJ-3022)-against postherpetic neuralgia aggravated by self-reported cold stimulation: a case series. J Altern Complement Med. 2012;18:686-92.
6. Aki T, Mihara M. Treatment for herpes-zoster associated pain with oriental herbal medicines. Nishinihon J Dermatol. 2002;64:363-6.
7. Nakamura Y, Tajima K, Kawagoe I, Kanai M, Mitsuhata H. Efficacy of traditional herbal medicine Yokukansan on patients with neuropathic pain. Jpn J Anesthesiol. 2009;58:1248-55.
8. Shukuwa T, Chen WY, Kobayashi A. Statistical analysis of herpes zoster observed at Sasebo City General Hospital. Nishinihon J Dermatol. 2002;64:477-82.
9. Kongkaew C, Chaiyakunapruk N. Efficacy of Clinacanthus nutans extracts in patients with herpes infection: systematic review and meta-analysis of randomised clinical trials. Complement Ther Med. 2011;19:47-53.
10. Sangkitporn S, Chaiwat S, Balachandra K, Na-Ayudhaya TD, Bunjob M, Jayavasu C. Treatment of herpes zoster with Clinacanthus nutans (bi phaya yaw) extract. J Med Assoc Thai. 1995;78:624-7.
11. Charuwichitratana S, Wongrattanapasson N, Timpatanapong P, Bunjob M. Herpes zoster: treatment with Clinacanthus nutans cream. Int J Dermatol. 1996;35:665-6.
12. Guo H, Mao J, Qian X, Sun C, Sun H. Varicella-zoster virus prophylaxis with the traditional Chinese medicine radix isatidis (Banlangen) in patients with multiple myeloma treated with bortezomib. J Altern Complement Med. 2011;17:985-6.
13. Peng Z, Wang S, Huang X, Xiao P. Effect of hyperbaric oxygen therapy on patients with herpes zoster. Undersea Hyperb Med. 2012;39:1083-7.
14. Byun SH, Jeon Y. Administration of vitamin C in a patient with herpes zoster - A case report -. Korean J Pain. 2011;24:108-11.
15. Lee SM, Lim J, Lee JD, Choi DY, Lee S. Bee venom treatment for refractory postherpetic neuralgia: a case report. J Altern Complement Med. 2014;20:212-4.
16. Jung W, Lee BJ, Kim SS, Lee YJ. The effect of Gunn's intramuscular stimulation for postherpetic neuralgia -A report of 4 cases-. Korean J Anesthesiol. 2010;58:311-7.
17. Yu XM, Zhu GM, Chen YL, Fang M, Chen YN. Systematic assessment of acupuncture for treatment of herpes zoster in domestic clinical studies. Zhongguo Zhen Jiu. 2007;27:536-40.
18. Fang X. Clinical observation of acupuncture therapy for postherpetic neuralgia. J Acupunct Tuina Sci. 2011;9:113-5.
19. Ni XL. Treatment of 48 cases of herpes zoster by puncturing nerve stem. J Acupunct Tuina Sci. 2008;6:368-9.
20. Cao H, Li X, Liu J. An updated review of the efficacy of cupping therapy. PLoS One. 2012;7:e31793.
21. Cao H, Han M, Li X, Dong S, Shang Y, Wang Q, et al. Clinical research evidence of cupping therapy in China: a systematic literature review. BMC Complement Altern Med. 2010;10:70.
22. Chen B, Gao C, Li C, Chen ZL, Guo Y. Discussion on the suitable diseases and symptoms of bleeding therapy. Zhongguo Zhen Jiu. 2009;29:397-9.
23. Wang X. Treating different diseases with the point ear apex. J Chin Med. 2006;(82):47-8.
24. Zhang B. Observation in the treatment of herpes zoster by far-infrared radiator (TDP). Hongwai Jishu. 1994;16:35-6.
25. Huang B, Zhou XY, Lu YP, Zhu ZF, Hou J, Sun JL, et al. Selective percutaneous dorsal root ganglion radiofrequency thermocoagulation guided by CT scanning in treatment of post-herpetic neuralgia. Nat Med J China. 2008;88:885-8.

26. Mittal RR, Jassal JS, Bahl RK. Laser therapy in post herpetic neuralgia. Indian J Dermatol Venereol Leprol. 1996;62:229-30.

27. Li ZY, Kuang GP, Zhu JD, Wang HY, Zhu MQ, Wu ZQ. Clinical effect of He-Ne laser combined with medicine therapy on herpes zoster keratitis. Intern Eye Sci. 2012;12:1922-4.

28. Moriyama K. Effect of temporary spinal cord stimulation on postherpetic neuralgia in the thoracic nerve area. Neuromodulation. 2009;12:39-43.

29. Jung SW, Son HH, Lee YS, Lee SK. The effect of light emitting diode on acute pain and the inhibitory effects of postherpetic neuralgia in patients with herpes zoster. Korean J Dermatol. 2013;51:763-70.

30. Park KY, Han TY, Kim IS, Yeo IK, Kim BJ, Kim MN. The effects of 830 nm light-emitting diode therapy on acute herpes zoster ophthalmicus: A pilot study. Ann Dermatol. 2013;25:163-7.

31. Lim SM, Park HL, Moon HY, Kang KH, Kang H, Baek CH, et al. Ultrasound-guided infraorbital nerve pulsed radiofrequency treatment for intractable postherpetic neuralgia - a case report -. Korean J Pain. 2013;26:84-8.

32. Ko YK, Lee HY, Lee WY. Clinical experiences on the effect of scrambler therapy for patients with postherpetic neuralgia. Korean J Pain. 2013;26:98-101.

33. Huo HM, Yang XP. Observation on therapeutic effect of pricking blood therapy combined with acupuncture on herpes zoster. Zhongguo Zhen Jiu. 2007;27:729-30.

34. Pan H. Observation of curative effect of herpes zoster treated with acupuncture based on syndrome differentiation combined with pricking and cupping. Zhongguo Zhen Jiu. 2011;31:901-4.

35. Li XW, Yang YK, Xie XM, Bai LN, Zhang XS. Economic evaluation of treating herpes zoster with various methods of acupuncture and moxibustion. J Tradit Chin Med. 2012;32:125-8.

36. Huang GF, Zhang HX, Xu ZS, Li JW. Comparison of therapeutic effects of different types of acupuncture interventions on herpes zoster in acute stage. Zhen Ci Yan Jiu. 2012;37:403-8.

37. Yu F, Xu SW, Zhang W. Clinical observation on treatment of herpes zoster with collateral-pricking and healthy energy strengthening therapy. Zhong Xi Yi Jie He Xue Bao. 2005;3:400-1.

38. Zhang M, Qiu L, Zhang J. Observation on therapeutic effect of surround needling plus surround moxibustion on herpes zoster. Zhongguo Zhen Jiu. 2007;27:123-5.

39. Wang S. Treatment of 30 cases of post-herpetic neuralgia by acupuncture combined with point injection. J Acupunct Tuina Sci. 2008;6:182-3.

40. Tian HY, Hu J, Yang JB. Comparison on therapeutic effect between surround needling plus thin cotton moxibustion and Western medicine for herpes zoster. Zhongguo Zhen Jiu. 2011;31:219-22.

41. Yang JX, Xiang KW, Zhang YX. Treatment of herpes zoster with cotton sheet moxibustion: multicentral randomized controlled trial. Zhongguo Zhen Jiu. 2012;32:417-21.

42. Han L, Zhao S. Zoster and postherpetic neuralgia treated by percussopunctato combined with cupping therapy 33 cases. Chin J Clin Rehab. 2003;7:699.

43. Wu B, Jiang CH, Zhou QY, Chen QM, Shu Y, Li X. Treatment of residual neuralgia of herpes zoster by ear point taping and pressing therapy combined with acupoint-injection. Zhongguo Zhen Jiu. 2007;27:807-9.

44. Ouyang Q, Wei ZJ, Hou YL. Pricking blood therapy combined with ultraviolet irradiation for treatment of acute herpes zoster. Zhongguo Zhen Jiu. 2009;29:285-8.

45. Li X, Zhang HX, Huang GF, Feng YF, Zou R. Observation on the therapeutic effect of electroacupuncture of Jiaji (EX-B 2) plus regional encircled needling for herpes zoster. Zhen Ci Yan Jiu. 2009;34:125-7, 135.

46. Liu YN, Zhang HX, Huang GF, Zou R, Wei W. Observation on therapeutic effect of electroacupuncture at Jiaji (EX-B 2) combined with blood-letting and cupping on herpes zoster. Zhongguo Zhen Jiu. 2009;29:887-90.

47. Liu YN, Zhang HX, Huang GF, Zou R, Wei W. Treatment of herpes zoster with electroacupuncture plus collateral-pricking and cupping therapy. J Acupunct Tuina Sci. 2013;11:282-5.

48. Hu J. Acupuncture treatment of herpes zoster. J Tradit Chin Med. 2001;21:78-80.

49. Dai J, Yin AH, Zhou Y, Yin LJ. Herpes zoster treated with meridian-collateral electric information therapy combined with pricking and cupping. Zhongguo Zhen Jiu. 2011;31:416-9.

50. Wang ZJ, Zou Y. Observation on therapeutic effects of electroacupuncture at Jiaji (Ex-B 2) points plus herbal medicine for herpes zoster. J Acupunct Tuina Sci. 2012;10:313-7.

51. Fan YJ, Liu QG, Yin GZ. Therapeutic observation on swift needling with fire needle plus medication for herpes zoster. J Acupunct Tuina Sci. 2013;11:380-3.

52. Guo LH, Chen X, Huang P, Liang YC, Mu JP. Clinical study on Fu's subcutaneous needling with laser for postherpetic neuralgia. J Acupunct Tuina Sci. 2014;12:165-8.

53. He YD, Fang RH. Treatment of 60 cases of senile herpes zoster by encircled acupuncture plus valaciclovir. J Acupunct Tuina Sci. 2007;5:171-3.

54. Song HY, He HQ. Clinical study on surrounding acupuncture for herpes zoster. J Acupunct Tuina Sci. 2013;11:278-81.

55. Tian ZW, Song XF, Li WL, Feng J. Chinese medicine plus mecobalamin for postherpetic neuralgia in patients with refractory herpes zoster. Chin J Clin Rehab. 2005;9:24-5.

56. Wang XP, Mok MS, Li YI, Cai JY. Combined therapy of Super Lizer and Durogesic patch in elderly patients with herpes zoster and diabetes mellitus. J Clin Rehab Tissue Eng Res. 2007;11:2589-92.

57. Wang CY, Fang JQ. Analysis on therapeutic effect of variable-frequency electroacupuncture combined with herbal-moxa moxibustion for post-zoster neuralgia. Zhen Ci Yan Jiu. 2012;37:64-6.

58. Xie JH. The quality of life of post herpes zoster neuralgia. Chin J Clin Rehab. 2003;7:3610-11.
